# Non-oxide precipitates in additively manufactured austenitic stainless steel

**DOI:** 10.1038/s41598-021-89873-2

**Published:** 2021-05-17

**Authors:** Manas Vijay Upadhyay, Meriem Ben Haj Slama, Steve Gaudez, Nikhil Mohanan, Lluis Yedra, Simon Hallais, Eva Héripré, Alexandre Tanguy

**Affiliations:** 1 Laboratoire de Mécanique des Solides (LMS), CNRS UMR 7649, Ecole Polytechnique, Institut Polytechnique de Paris, 91128 Palaiseau, France; 2 Laboratoire de Mécanique des Sols, Structures et Matériaux (MSSMat), CNRS UMR 8579, CentraleSupélec, Université Paris-Saclay, 91190 Gif-sur-Yvette, France; 3 Institut Jean Lamour (IJL), CNRS UMR 7198, Université de Lorraine, Campus ARTEM, Nancy, France; 4 Laboratoire Structures, Propriétés et Modélisation des Solides (SPMS), CNRS UMR 8580, CentraleSupélec, Université Paris Saclay, 91190 Gif-sur-Yvette, France; 5 Present Address: Department of Electronics and Biomedical Engineering, Institute of Nanoscience and Nanotechnology (IN2UB), University of Barcelona, 08028 Barcelona, Catalonia Spain

**Keywords:** Metals and alloys, Engineering

## Abstract

Precipitates in an austenitic stainless steel fabricated via any Additive Manufacturing (AM), or 3D printing, technique have been widely reported to be only Mn-Si-rich oxides. However, via Transmission Electron Microscopy (TEM) studies on a 316L stainless steel, we show that non-oxide precipitates (intermetallics, sulfides, phosphides and carbides) can also form when the steel is fabricated via Laser Metal Deposition (LMD)—a directed energy deposition-type AM technique. An investigation into their origin is conducted with support from precipitation kinetics and finite element heat transfer simulations. It reveals that non-oxide precipitates form during solidification/cooling at temperatures ≥ 0.75T_m_ (melting point) and temperature rates ≤ 10^5^ K/s, which is the upper end of the maximum rates encountered during LMD but lower than those encountered during Selective Laser Melting (SLM)—a powder-bed type AM technique. Consequently, non-oxide precipitates should form during LMD, as reported in this work, but not during SLM, in consistency with existing literature.

## Introduction

Additive Manufacturing (AM), or 3D printing, of alloys is a revolutionary technology that simultaneously manufactures a part with a desired geometry and creates the material microstructure without the need for tooling. This unique ability has generated a widespread industrial interest to adopt AM not only to manufacture entire alloy parts but also to repair broken/damaged parts. However, manufacturing entire parts or repairing portions of a broken part via AM to obtain microstructures that exhibit desired material properties is a great challenge, and it has been a subject of intensive research in recent years.

An AM process involves reading a 3D part geometry from a computer-aided design file and building it in a layer-by-layer manner by locally melting a feedstock (powder/wire) using a moving heat-source (laser/electron beam). Based on the manner in which heat-source and feedstock interactions occur, alloy AM processes can be classified^1^ into (i) Directed Energy Deposition (DED), where the feedstock material is directly fed into the moving heat-source, and (ii) Powder Bed Fusion (PBF), a two-step repetitive process involving powder deposition on a powder-bed followed by scanning via a heat-source. AM of austenitic stainless steel parts is typically done via Laser Metal Deposition (LMD: a laser-based DED approach) and Selective Laser Melting (SLM: a laser-based PBF approach). In both approaches, the heat-matter interactions subject the material to a sequence of highly non-equilibrium processes, viz. melt-pool dynamics, rapid solidification and solid-state cooling-heating cycles, which result in the formation of hierarchical microstructures exhibiting physical and chemical heterogeneities at multiple length scales^1^.

In the case of 316L Stainless Steel (316LSS) fabricated via SLM or LMD, the hierarchical microstructures are composed^1–8^ of (i) precipitates, micro-segregations, porosities and dislocation structures at the intragranular level and (ii) heterogeneous grain morphologies and sizes, texture and voids at the polycrystalline level. Amongst all these features, precipitates are one of the smallest in size and they play a crucial role in determining the material response^3,4^. During plastic deformation, precipitates can impede dislocation motion resulting in hardening via mechanisms such as Orowan bypass, cross-slip, etc.^9^ Furthermore, the presence/absence of some precipitates can affect the corrosion, wear, fatigue and fracture resistance of a steel^2,10^. It is therefore important to understand their origin in order to control their formation in the microstructure and optimize material properties.

Conventionally processed/post-processed 316LSS is known to contain a plethora of different kind of precipitates including oxides of transition metals (Fe, Cr, Ni, Mn, Mo) and silicon^11–13^ as well as non-oxides such as transition-metal carbides^10^, sulfides^14–17^, phosphides^18^ and intermetallics^19,20^. In contrast, however, a series of recent Transmission Electron Microscopy (TEM) studies^2–7^ report the sole presence of Mn-Si–O precipitates in SLM and LMD 316LSS. This significant difference in the type of precipitates between conventionally processed/post-processed and additively manufactured (LMD or SLM) 316LSS can be attributed to the differences in the temperature rates encountered during these processes. In addition, oxidation of Mn and Si precipitates results in the highest reduction in the Gibbs free energy in comparison to other transition-metal (Fe, Cr, Ni and Mo) oxides^21^, which favors the formation of Mn-Si-O over other oxide precipitates.

It is well known that precipitate sizes in steels decrease with increasing solidification/cooling temperature rates^15,16^. For conventionally processed/post-processed 316LSS, where temperature rates much less than 10^2^ K/s are encountered, oxide and non-oxide precipitate sizes typically range from 1 μm to several tens of microns^15,16^. Meanwhile, during LMD and SLM processes, the maximum temperature rates encountered fall in the range 10^2^–10^5^ K/s^22^ and 10^6^–10^7^ K/s^23^, respectively. These magnitudes are consistent with the average size of Mn-Si-O precipitates reported in LMD 316LSS (> 100 nm) and SLM 316LSS (< 100 nm)^3,7^, respectively. However, none of these works report the presence of non-oxide precipitates. Based on these studies, one could deduce that the temperature rates at which non-oxide precipitates stop forming in 316LSS should be below 10^2^ K/s, which is the lower end of the maximum rate encountered during any AM process.

Recent TEM studies on 316LSS powders fabricated via inert gas atomization^1,7,24^ have revealed the presence of a significant amount of non-oxide precipitates (rich in Mo, Cr, P and S) together with Mn-Si–O precipitates. Interestingly, the maximum cooling rates encountered during inert gas atomization^25–27^ are in the same range as those occurring during LMD i.e., 10^2^–10^5^ K/s, which suggests that there should be a significant presence of non-oxide precipitates in LMD 316LSS. However, none of the existing studies on precipitates in LMD 316LSS conclusively report the presence of non-oxides. In a very recent study, Barkia et al.^28^ reported the presence of an Mo-Cr-rich zone and an Mn–Mo–Cr-rich zone (around an Mn–Si–O precipitate) within the walls of an intragranular cellular solidification structure in their LMD 316LSS. However, these cell walls are known to be sites of preferential segregation of Mo and Cr^1^, and the reported presence of Mo–Cr and Mn–Mo–Cr zones can be considered only as circumstantial evidence of the presence of non-oxide precipitates.

In light of the above, the main aim of this paper is to answer the following questions: Can non-oxide precipitates form during LMD of 316LSS? What is the threshold temperature and temperature rate above which non-oxide precipitates stop forming in 316LSS? To answer the first question, we have performed a series of Scanning-TEM (STEM) studies together with Electron-Dispersive X-ray Spectroscopy (EDS). To answer the second question, we have performed Finite Element (FE) heat transfer simulations to better understand the temperature rates encountered during LMD and used this information together with the results of precipitation kinetics simulations to understand the role of temperature and temperature rates on nucleation and growth of non-oxide precipitates. This analysis is also applied to understand the absence of non-oxides in SLM 316LSS.

The 316LSS powder used in this work had been produced via the inert gas atomization process by Höganäs AB. The wrought alloy used to manufacture this powder had the chemical composition in weight percent (wt. %): Cr—16*.*9, Ni—12.7, Mo—2.5, Mn—1.5, Si—0.7, P—0.015, C—0.011, S—0.005 and Fe-balance. Presence of trace amounts of oxygen in the inert gas atomization chamber can introduce oxygen into the 316LSS powder obtained at the end of this process; according to the documentation of Höganäs AB^29^, a 316LSS powder particle produced via their inert gas atomization process can contain up to 0.05 wt. % of O.

**Figure 1:**
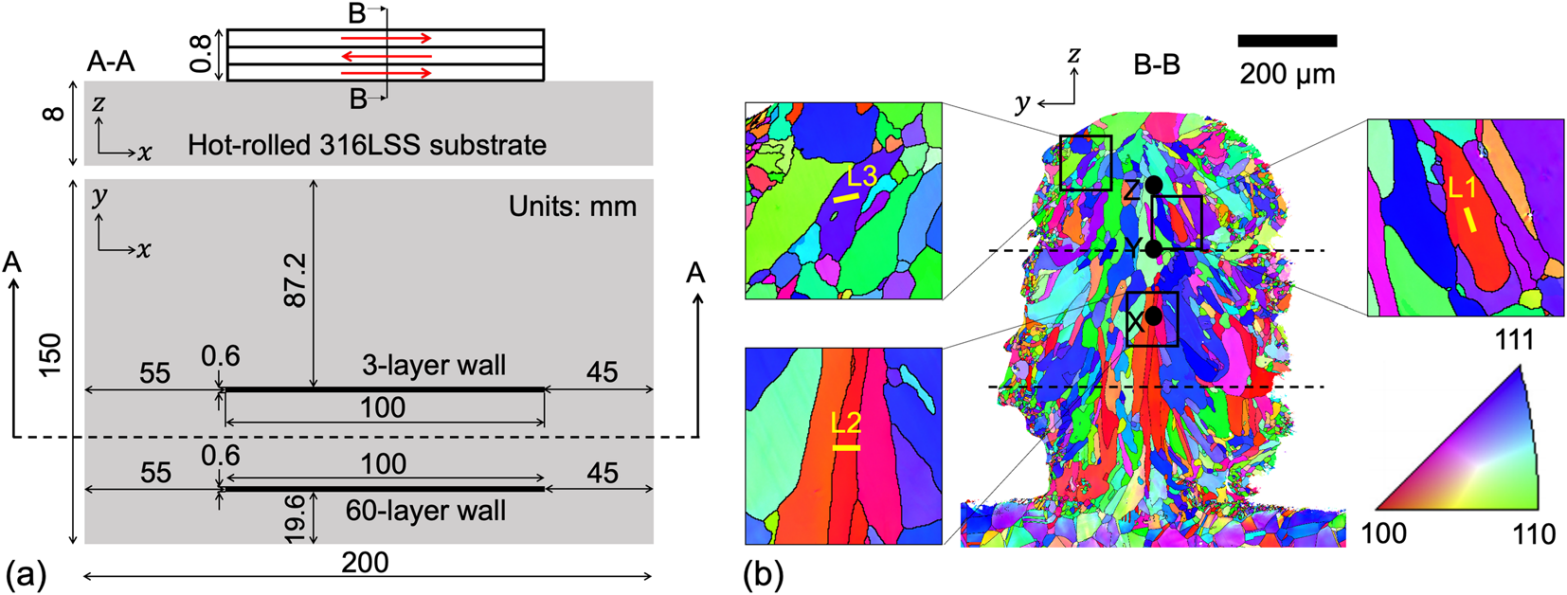
Design and crystallographic orientation map of the LMD 316LSS walls from which TEM lamellae are extracted. **(a)** An illustration (not-to-scale) of the single-track bidirectionally-printed 3-layer and 60-layer LMD 316LSS walls on a hot-rolled 316LSS substrate. Directions $x$, $y$ and $z$ respectively represent the printing, thickness and building directions. The top view and the front view of a cross-section A–A are shown. The red arrows in the front-view show the direction of printing of each layer. **(b)** An EBSD orientation map color-coded according to IPF along building direction at a cross-section B–B approximately at the mid-section of the 3-layer wall. Dotted lines in **(b)** represent the approximate position of interlayer boundaries. The black solid circles in **(b)** indicate the points X, Y and Z that correspond to the locations in the FE simulations from where the temperature vs time data has been extracted and plotted in Fig. 7. Subfigures in **(b)** show zoomed-in EBSD maps. Yellow-colored lines demark zones underneath which lamellae L1, L2 and L3 are extracted (in the out-of-plane direction). These lines correspond to the top side of L1, L2 and L3 in the HAADF images in Fig. 2. Aztec v4.2 (Oxford Instruments https://nano.oxinst.com/products/aztec/) and Microsoft PowerPoint have been used to prepare this figure.

The 316LSS powder had been fed into the LMD machine to print a single-track (one linear laser pass per layer) 3-layer wall on a hot-rolled 316LSS substrate via a bidirectional (forth–back–forth) scanning strategy as illustrated in Fig. 1a. Following the building of the 3-layer wall and after the substrate had cooled down, a 60-layer wall was built on the same substrate as shown in Fig. 1a. The Electron Back-Scattered Diffraction (EBSD) orientation map of a cross-section in the middle part of the 3-layer wall (Fig. 1b) and the 60-layer wall (Fig. S1) reveal a weakly-textured microstructure with columnar grains in the bulk surrounded by a thin layer of smaller equiaxed grains, which is typical for an LMD process.

**Figure 2:**
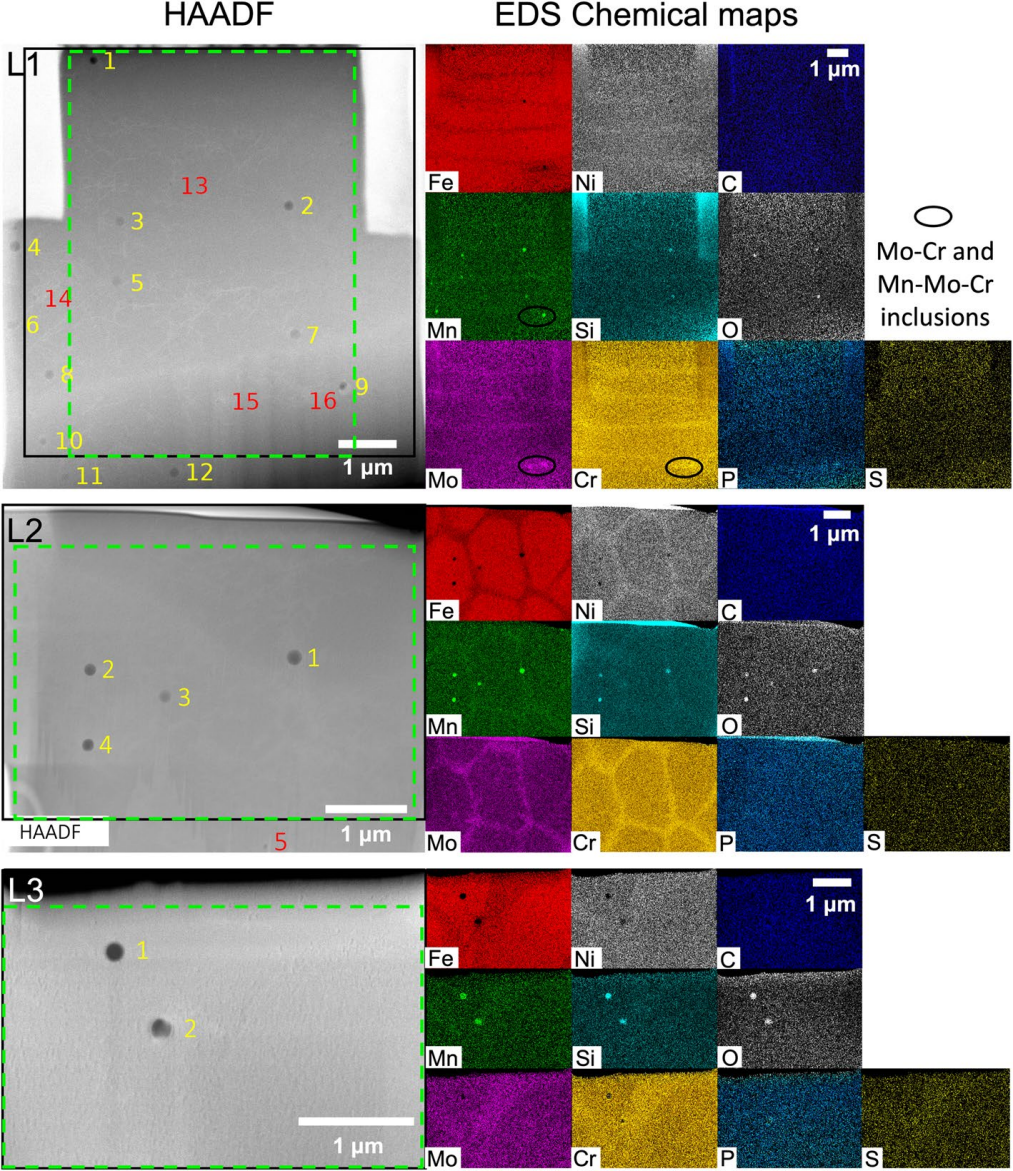
STEM HAADF images and EDS chemical maps of L1, L2 and L3 extracted from the 3-layer wall of Fig. 1. EDS maps have been generated from within the black outlined region in each HAADF image. The dotted green lines in each HAADF image represents the zone where the lamella composition has been computed and presented in Table S1 (Supplementary Data). Precipitates that are easily visible in the HAADF images are numbered in yellow font. Precipitates that have been detected after high resolution imaging are numbered in red font. Scale for EDS maps of each lamella are shown in their C map. HAADF images and EDS maps have been acquired using the TIA v4.2 (FEI https://www.fei.com) and the Esprit v1.9 (Brucker https://www.bruker.com) software, respectively. Fiji v2.1.0/1.53c (https://fiji.sc/) and Microsoft Powerpoint have been used to prepare this figure.

Three TEM lamellae (L1, L2 and L3), each thinner than 100 nm, had been extracted from three different columnar grains of cross-section A-A of the 3-layer wall (Fig. 1b). Two lamellae (L4 and L5) were extracted from two columnar grains, one near the top and one near the bottom of the 60-layer wall (Fig. S1). All lamellae were studied via (i) High-Angle Annular Dark-Field (HAADF) STEM imaging to identify the location of precipitates and (ii) EDS chemical mapping to understand their chemical composition (Fig. 2 and Fig. S2). HAADF images and EDS chemical maps help identify intragranular chemical heterogeneities; in LMD 316LSS, these heterogeneities are cellular solidification structures and precipitates.

Intragranular cellular solidification structures in any AM 316LSS typically have cell walls with lower concentration of Fe and higher concentration of Cr and Mo than the surrounding matrix^1,4^; these cellular structures can be discerned from the EDS maps in Fig. 2 and Fig. S2. These structures also have their long-axes aligned with the crystal growth direction during solidification^1^. Since L1 and L2 had been extracted in directions nearly parallel and perpendicular to the growth direction of their respective grains (Fig. 1), the long-axis of cellular structures in L1 and the grid-like cross-section of the cellular structures in L2 can be discerned. L3 forms a random angle with the grain growth direction, which makes it difficult to discern the cellular structures. Nevertheless, a Y-shaped band can be observed in its Fe, Cr and Mo maps. L4 also shows a complex intragranular cellular structure whose precise orientation is hard to discern from the Fe, Cr and Mo maps (Fig. S2). L5 has the long-axes of its cellular solidification structures aligned in-plane at a small angle with the horizontal direction. Similar to Barkia et al.^28^, Mo-Cr and Mo-Cr-Mn rich zones are observed in the cellular solidification structures in L1 and L4, however, at the magnification level shown in Fig. 2 and Fig. S2, it is not possible to discern whether or not these zones are forming precipitates.

The chemical composition of the lamellae L1–L5 obtained from the EDS maps shows that all lamellae have a lower wt. % of Fe and Cr and higher wt. % of Mn, O, P, S and C than the wrought alloy (Table S1). Ni is higher for L1, L2, L4 and L5 but lower for L3. Mo is higher for L3 and L5 but lower for the rest. Si is higher for L1, L2 and L3 but lower for the rest.

**Figure 3:**
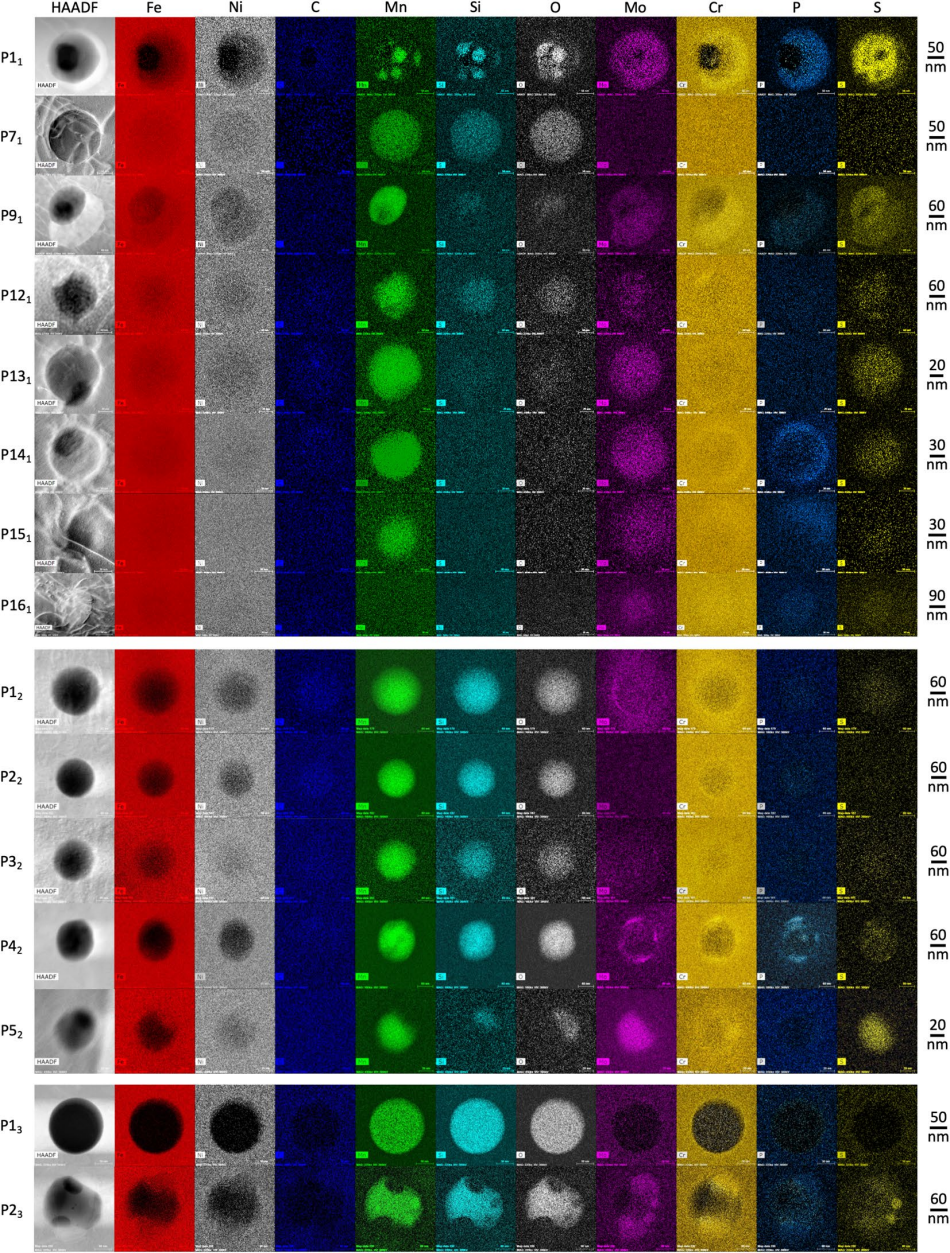
STEM HAADF images and EDS maps of all non-oxide and mixed precipitates, and at maximum one oxide precipitate, in L1–L3. To facilitate visualization, picture corrections have been made for all the EDS maps. ImageJ and Microsoft PowerPoint have been used to prepare this image. HAADF images and EDS maps have been acquired using the TIA v4.2 (FEI https://www.fei.com) and the Esprit v1.9 (Brucker https://www.bruker.com) software, respectively. Fiji v2.1.0/1.53c (https://fiji.sc/) and Microsoft PowerPoint have been used to prepare this figure.

55 precipitates have been identified in these lamellae: 16 in L1, 5 in L2, 2 in L3, 17 in L4 and 15 in L5. Most of them could be discerned from the HAADF images in Fig. 2 and Fig. S2, however, some of them were discovered after performing high-resolution HAADF imaging (Fig. 3 and Fig. S3). In order to facilitate identifying the precipitates and differentiating between them, a classification scheme and a nomenclature system is proposed in Table 1. Based on this classification scheme, out of the 55 precipitates, 16 are oxides (29.1%), 3 are non-oxides (5.4%) and 36 (65.5%) are mixed.

**Table 1:** Classification and nomenclature of precipitates in lamellae L1–L5.

Precipitate classification	Description	Nomenclature				
		L1	L2	L3	L4	L5
Oxide	Single inclusion with nearly uniform composition. Contains higher O than the matrix and appears darker than matrix in the HAADF image	P2_1_–P8_1_, P10_1_, P11_1_	P3_2_	P1_3_	P1_4_, P11_4_	P8_5_, P14_5_, P15_5_
Non-oxide	Single inclusion with nearly uniform composition. Contains lesser oxygen than the matrix and appears brighter than oxide precipitates in HAADF images	P16_1_	–	–	P15_4_	P7_5_
Mixed	Two or more inclusions with different elemental compositions	P1_1_, P9_1_, P12_1_–P15_1_	P1_2_, P2_2_, P4_2_, P5_2_	P2_3_	P2_4_–P10_4_, P12_4_–P14_4_, P16_4_, P17_4_	P1_5_–P6_5_, P9_5_–P13_5_

Precipitates are classified into oxides, non-oxides and mixed based on the oxygen content (in EDS maps) and brightness (in HAADF image) with respect to the surrounding (austenite) matrix. Each precipitate is numbered according the sequence shown in the HAADF images in Fig. 2 and the lamella number is added as a subscript.

**Figure 4:**
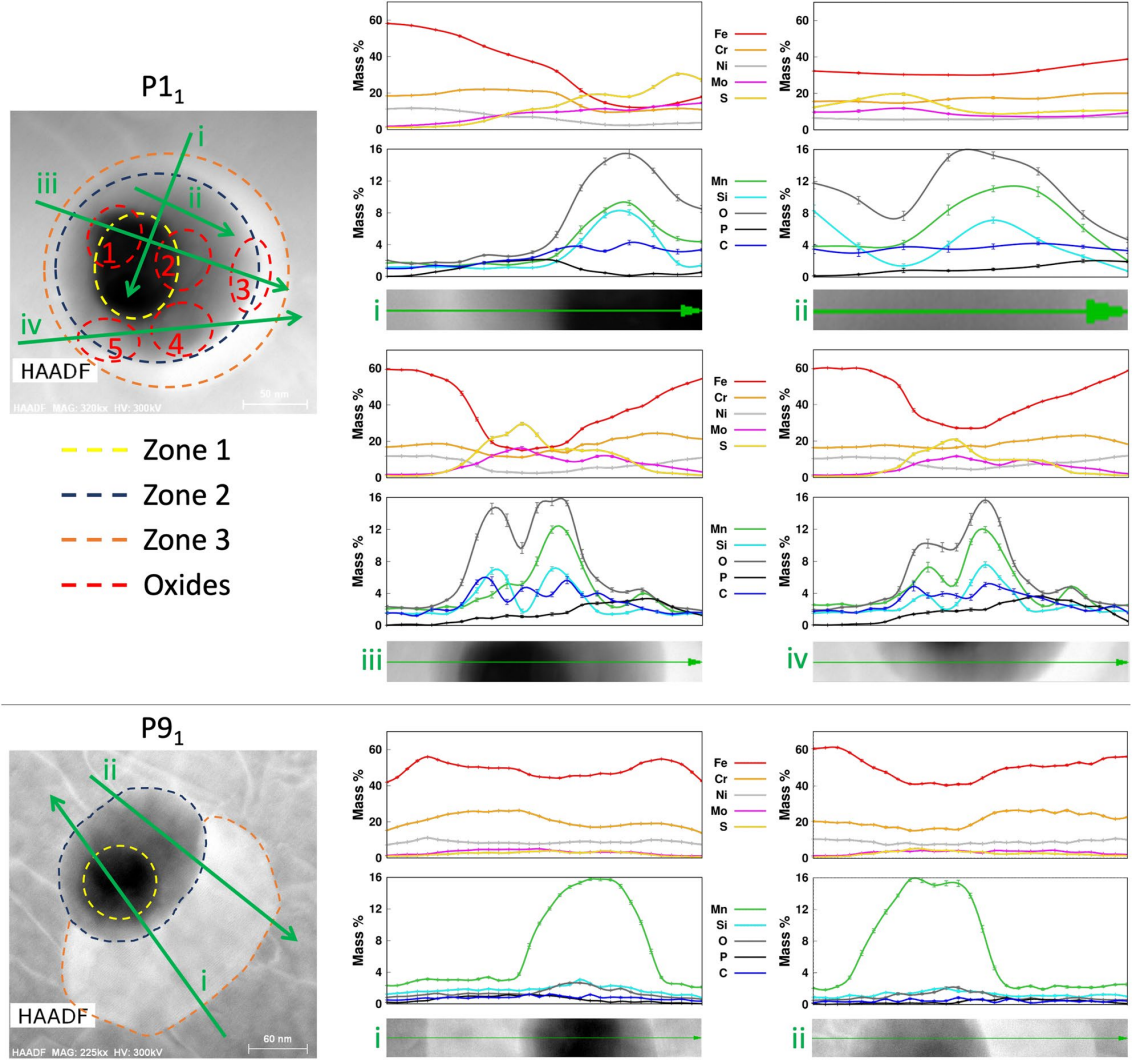
HAADF images and EDS line profiles for precipitates P1_1_ and P9_1_ along different paths. The points in the curves have been fitted using c-splines. The key for both HAADF images is presented below the HAADF image of P1_1_. HAADF images have been acquired using the TIA v4.2 (FEI https://www.fei.com) software. Fiji v2.1.0/1.53c (https://fiji.sc), gnuplot v5.4 (http://www.gnuplot.info) and Microsoft PowerPoint have been used to prepare this image.

Figure 3 shows the HAADF images and EDS chemical maps for all non-oxide and mixed precipitates together with at most one oxide precipitate (if any) in L1–L3; Fig. S3 shows the HAADF images and EDS maps for non-oxide and mixed precipitates in L4 and L5. EDS line profile analysis (Methods) has been performed for each precipitate; Fig. 4 shows the EDS line profiles for two of the most complex mixed precipitates. Following this analysis, the chemical composition of all precipitates had been compared with the nominal composition of the 316LSS wrought alloy. Based on this comparison the elements in each oxide and non-oxide precipitate, and all the inclusions in each mixed precipitate, have been classified into two categories: (i) higher and (ii) lower composition than wrought alloy. This classification is used to tabulate each element in all precipitates of L1, L2 and L3 in Table S1.

All precipitates consistently show a decrease in Fe with respect to the wrought alloy and the lamella to which they belong. Most precipitates show a decrease in Ni, however, these concentrations never become zero. TEM lamella preparation can lead to partial slicing of precipitates and in most cases include some proportion of the surrounding matrix along the thickness direction; this is also evidenced via the distortions caused by dislocation lines in the HAADF images of oxides (Fig. 3). Hence, non-zero compositions of Fe in all precipitates should mainly arise from the matrix above or below the precipitate.

All oxides are amorphous in nature, which has been confirmed from high-resolution STEM imaging and convergent beam diffraction (not shown) and it is consistent with existing literature^7^. They are richer in Mn–Si–O with respect to the wrought alloy, however, in some cases, Si and O are lower in wt. % than their respective compositions in the associated lamellae (Table S1). Furthermore, proportions of Mn, Si and O vary (drastically in some cases) from one oxide to another. These results indicate the presence of different combinations of Mn_x_Si_y_O_z_ (x,y ≥ 0, z > 0) in these precipitates. Along with Mn-Si-O, oxides have higher concentrations of P, S and C than the wrought alloy but often lower C concentration than their corresponding lamella (Table S1); furthermore, in most cases, the P, S and C concentrations increase from the oxide center to the oxide-matrix interface.

Amongst the 3 non-oxide precipitates, (i) P16_1_ is richer in Cr–Mo–Mn–Si–O–P–S–C in comparison to the wrought alloy and L1, (ii) P15_4_ is richer in Ni–Mo–Mn–O–P–S–C in comparison to the wrought alloy but only Mo-Mn-P-S are higher than those in L4, and (iii) P7_5_ is richer in Ni–Mo–Mn–O–P–S in comparison to the wrought alloy but only Mo-Mn-P are higher than those in L5. All non-oxide precipitates are embedded either above, below or in between the matrix. Whenever a non-oxide precipitate contains a higher wt. % of Cr than its surroundings, it results in a brighter contrast with respect to its surrounding in the HAADF image, as evidenced from P16_1_.

Similar to non-oxide precipitates, all mixed precipitates also exhibit a non-zero oxygen composition with respect to the wrought alloy, and all non-oxide inclusions in mixed precipitates that have a higher wt. % of Cr than the surrounding matrix show a brighter contrast in the HAADF images. Out of the 36 mixed precipitates, 34 (94.44%) contain both oxide and non-oxide inclusions; P15_1_ and P14_4_ contain only non-oxide inclusions. Amongst these 34 mixed precipitates, 26 (72.22% out of 36) clearly show oxide inclusions at their core and non-oxide inclusions around the oxides. However, it is harder to arrive at this conclusion for the remaining 8 (22.22% out of 36): P1_1_, P9_1_, P3_4_, P8_4_, P12_4_, P1_5_, P2_5_ and P4_5_.

Out of the 36 mixed precipitates, 30 are composed of two inclusions each that manifest either next to each other or one surrounds the other; they are P12_1_–P15_1_, P1_2_, P2_2_, P4_2_, P5_2_, P2_4_, P4_4_–P6_4_, P8_4_–P10_4_, P13_4_, P14_4_, P16_4_, P17_4_, P1_5_–P6_5_, P9_5_–P13_5_. The remaining 6 i.e., P1_1_, P9_1_, P2_3_, P3_4_, P7_4_ and P12_4_ are composed of more than 2 inclusions.

P1_1_ is a complex combination of 3 zones and 5 small inclusions. Based on the morphology, brightness evolution and composition of these zones, we can deduce that (i) zone 1 was part of a larger oxide inclusion before the lamella was prepared, (ii) zone 2 is made up of two inclusions: one is the same oxide as in zone 1 and the other is a Cr–Mo–P–S non-oxide that overlaps the first, (iii) the brightness changes across zone 2 as the amount of the oxide inclusion along L1’s thickness direction decreases and that of the non-oxide inclusion increases. (iv) The oxide terminates at the interface between zone 2 and zone 3 such that (v) zone 3 is rich only in Cr–Mo–P–S (Figs. 3 and 4). Due to the high O-content of the five elliptical inclusions, they are classified as oxides. They are also rich in Cr–Mo–Mn–P–S–C with respect to the wrought alloy but these contributions could arise due the overlap of these inclusions with zones 2 and 3 along L1’s thickness.

P9_1_ also has three zones. Similar to P1_1_, the morphology of these zones and of the surrounding matrix suggests that prior to L1’s preparation, zone 1 was part of a larger oxide inclusion and zone 2 includes a portion from the same oxide inclusion embedded underneath or above the surrounding matrix. The portion of this oxide in L1 terminates at the outer boundary of zone 2. Zone 3 is a non-oxide inclusion richer in Cr–Mo–P–S–C with respect to the wrought alloy and Cr–Mo–P–S with respect to L1.

The presence of different zones in P2_3_ can be explained in a manner similar to P1_1_ and P9_1_. However, the morphology of different inclusions, their composition and difference between their contrast in the HAADF image, clearly shows that the oxide inclusion in P2_3_ is not circular and it is highly unlikely that it would have been spherical prior to the extraction of L3. Distorted oxide inclusions are also observed in other mixed precipitates such as P12_1_. Meanwhile, pure oxide precipitates always manifest as circles. Therefore, these results strongly suggest that the presence of non-oxides can deviate the morphology of oxide inclusions away from a perfect spherical shape.

Similar to P1_1_, P3_4_ and P12_4_ also contain multiple small oxide inclusions embedded in non-oxide inclusions (Fig. S3); their sizes and distributions make it hard to separate them into clear zones. The oxide inclusions in P12_4_ are rich only in Mn–O; the composition of Si is lower than that in the wrought alloy and the surrounding matrix. P7_4_ contains a single large oxide inclusion at the core and it is surrounded by multiple non-oxide inclusions richer in Mo–Cr–P and Mo–Cr–S than the surrounding matrix. Meanwhile, contrary to other mixed precipitates containing oxide inclusions in the core, P8_4_, P1_5_, P2_5_ and P4_5_ contain a large non-oxide inclusion at their core surrounded by a smaller oxide inclusion.

P15_1_ and P14_4_ are the only mixed precipitates that are composed of only non-oxide inclusions. P15_1_ is made up of two inclusions next to each other. The darker one is richer in Ni–Mo–Mn–Si–O–P–S–C than the wrought alloy but only Mo–Mn–S–C are higher than in L1. The brighter one is also richer in Ni–Mo–Mn–Si–O–P–S–C than the wrought alloy but only Mo–Mn–P–C are higher than in L1. P14_4_ is made up of two nearly concentric non-oxide inclusions (Fig. S3). The larger one is richer in Ni-Mo-Mn–O–P–S than the wrought alloy but only Mo–P are higher than in L4. Interestingly, in comparison to the surrounding matrix, this inclusion is richer in Cr–Mo–P; the presence of Cr results in a brighter contrast with respect to the surrounding matrix in the HAADF image. The smaller darker one is richer in Mo–Mn–O–P–S than the wrought alloy but it is richer in Cr–Mo–Mn–P than L4. Interestingly, P14_4_ is the only precipitate that has negligibly small C composition with respect to the wrought alloy.

The TEM analysis has clearly shown that a plethora of different kinds of non-oxide inclusions are present in L1–L5. Furthermore, out of the 55 precipitates, 39 (70.9%) are either non-oxide precipitates or mixed precipitates containing only non-oxide inclusions. Bearing this fact in mind together with the fact that the TEM lamellae have been extracted from different locations of our LMD 316LSS walls and they occupy a very small volume in these walls, we can claim with high confidence that non-oxide inclusions are abundantly present in the 3-layer and the 60-layer LMD 316LSS walls.

**Figure 5:**
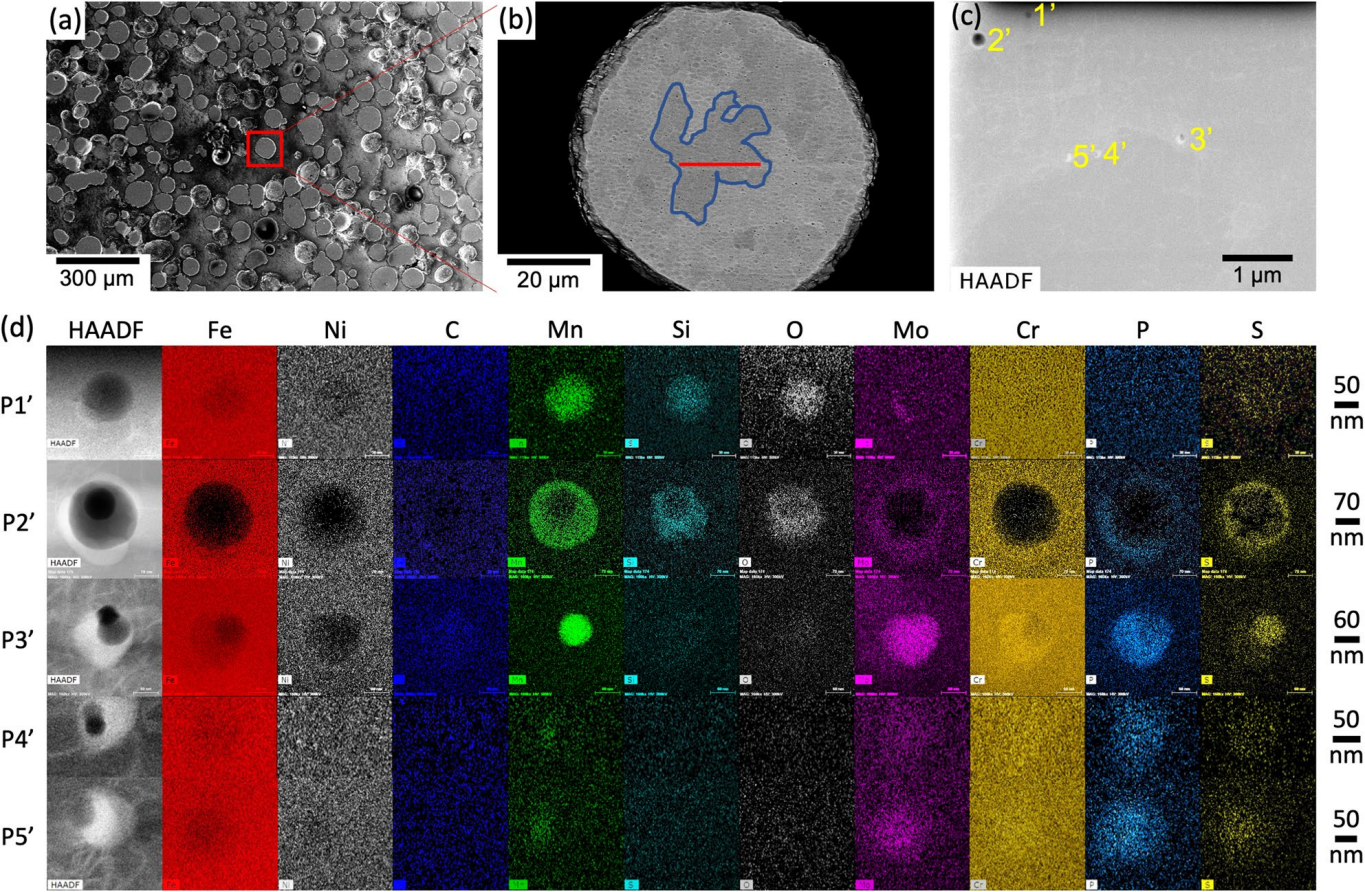
SEM micrograph of **(a)** polished 316LSS powder particles and **(b)** a powder particle at higher magnification. The TEM lamella LP in **(c)** is extracted from underneath the red zone in **(b)**, which is embedded within a grain whose outline is shown in blue. **(c)** STEM HAADF image of the lamella LP extracted from the 316LSS powder particle in **(b)**. **(d)** STEM HAADF images and EDS maps of all the precipitates of LP. Precipitates are named by adding a prefix “P” to their corresponding number in **(c)**. SEM micrographs have been captured using the XT v10 (ThermoFisher https://www.thermofisher.com/) software. HAADF images and EDS maps have been acquired using the TIA v4.2 (FEI http://www.fei.com) and the Esprit v1.9 (Brucker https://www.bruker.com) software, respectively. Fiji v2.1.0/1.53c (https://fiji.sc/) and Microsoft PowerPoint were used to prepare this figure.

In order to check whether similar kind of precipitates occur or not in the 316LSS powder, a TEM lamella “LP” had been prepared from a 316LSS powder particle (Fig. 5). The composition of LP is similar to L1–L5 (Table S1). It has lower Fe and Cr, slightly higher Ni, and the remaining alloying elements higher than the wrought alloy along with a high proportion of O. HAADF image of LP reveals 5 mixed precipitates containing both oxide and non-oxide inclusions with sizes similar to their counterparts in the 3-layer and 60-layer LMD 316LSS walls.

Now, the only common factor between inert gas atomization and LMD is the maximum temperature rate encountered during these processes, which is expected to be in the range 10^2^–10^5^ K/s^22,25–27^. Furthermore, it is well-known that the precipitate sizes in steels decrease with increasing temperature rate^15–17^. Based on this knowledge and the TEM results of this work, we hypothesize that the temperature rate encountered during AM of 316LSS is the main factor that determines the size of different kinds of precipitates. In order to verify this hypothesis, we have performed thermodynamics computations to study the precipitation kinetics of a non-oxide at different temperature rates. However, in order to perform these computations, we need (i) to assess the oxide and non-oxide inclusion/precipitate size distribution in our LMD 316LSS walls, and (ii) to estimate the temperature rates encountered during LMD of our 316LSS wall.

**Figure 6:**
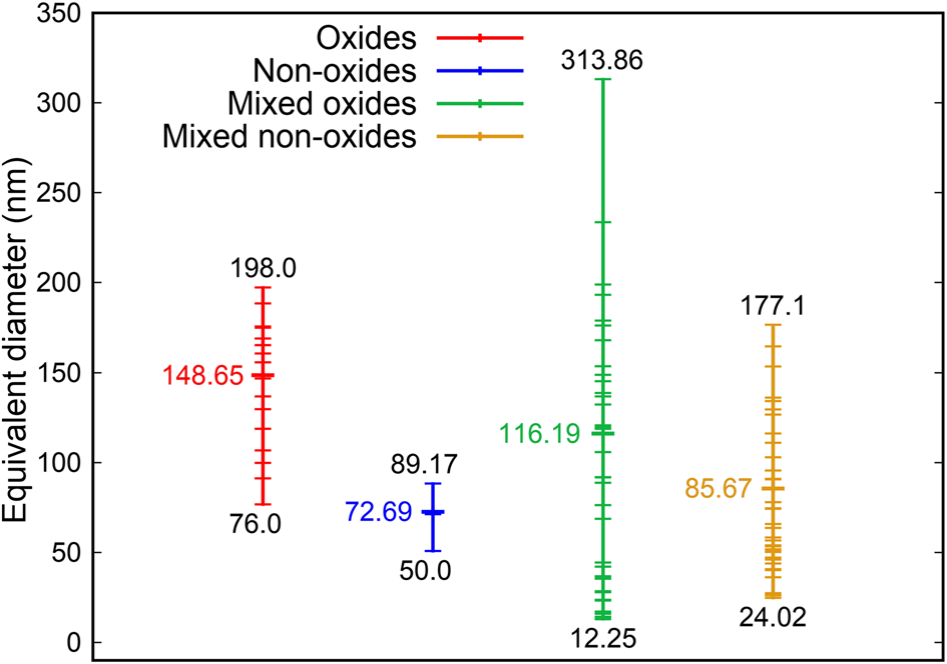
Comparison of equivalent diameters, also called size, of oxide precipitates (Oxides), non-oxide precipitates (Non-oxides), and the oxide (Mixed oxides) and non-oxide (Mixed non-oxides) inclusions in mixed precipitates in L1–L5. Equivalent diameter is the diameter of a circle whose area is equivalent to the corresponding area of the precipitate/inclusion in its HAADF image. Values in black font represent maximum and minimum equivalent diameters. Values in colored font represent average equivalent diameters, which are the diameters of a circle whose area is equivalent to the average of the areas of all precipitate/inclusions belonging to a category. The colored dashes represent the individual sizes of a precipitate/inclusion. Gnuplot v5.4 (http://www.gnuplot.info) was used to prepare this figure.

Figure 6 shows the size distribution of oxide and non-oxide precipitates and inclusions in L1–L5. In general, a wide scatter is observed in the size distribution of all precipitates and inclusions; the largest scatter occurs for oxide inclusions in mixed precipitates. The size distributions could be either (i) an apparent scatter caused by the 2D nature of a TEM analysis, or (ii) it could be due to differences in inclusion nucleation and growth rates arising from variations in local temperature rates. In order to diminish the influence of these effects, only the average sizes of the precipitates and inclusions are analyzed.

The average size of non-oxide precipitates/inclusions (72.69 nm/85.67 nm) is smaller than the average size of oxide precipitates/inclusions (148.65 nm/116.19 nm). A similar trend in the size differences between oxide and non-oxide inclusions is also found in the 316LSS powder. Furthermore, a similar trend has also been reported during solidification of a cast steel^15–17^. From here one can deduce that for the same temperature rate and starting temperature during processing of 316LSS, the average size of oxide inclusions/precipitates should be larger than the average size of their non-oxide counterparts irrespective of the processing technique used.

**Figure 7:**
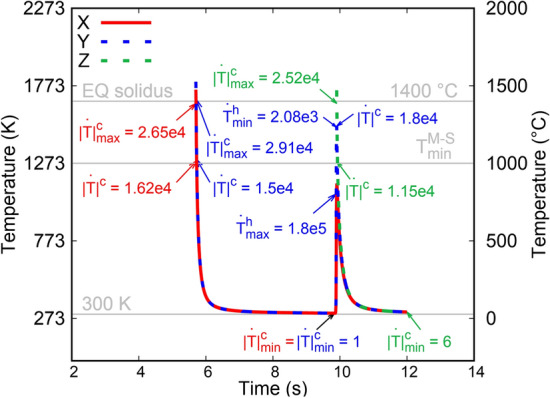
Temperature v/s time curves obtained from the heat transfer FE simulation of LMD of the 3-layer wall of Fig. 1b at the locations X, Y and Z. The temperature rates shown inside the plot have units K/s. Superscript ‘c’ implies cooling and ‘h’ implies heating. The rates in red, blue and green font correspond to the rates encountered by points X, Y and Z, respectively, during LMD. The subscripts ‘min’ and ‘max’ to the temperature rates correspond to minimum and maximum, respectively, rates for a given curve. EQ solidus stands for the equilibrium solidus temperature. ${\mathrm{T}}_{\mathrm{min}}^{\mathrm{M}-\mathrm{S}}$ is the minimum temperature below which M–S (M = Mn, Cr and Fe) does not precipitate for any temperature rate in the range 1–10^7^ K/s (see discussion related to Figs. 9 and 10). While the simulation begins at 0 s, however, X and Y are deposited at 5.7 s and Z is deposited at 9.9 s after the start. Gnuplot v5.4 (http://www.gnuplot.info) was used to prepare this figure.

In order to obtain an order of magnitude of the temperature rates that are encountered during the LMD of our samples, solid-state heat transfer FE simulations (see Methods for simulation setup) were performed for the 3-layer wall using the geometry shown in Fig. 1a. The temperature v/s time history was extracted from the mid-section of the 3-layer wall, approximately at the location of points X, Y and Z shown in Fig. 1b. These temperature v/s time curves are plotted in Fig. 7. Since points X and Y belong to the mid-layer of the 3-layer wall, they experience a cooling-heating-cooling sequence. Since Z belongs to the top layer of the 3-layer wall, it only experiences a cooling sequence. Just after deposition, the three points experience a temperature above the equilibrium solidus (1673 K) of 316LSS. However, since the FE simulations do not account for fluid dynamics or solidification, we shall restrict the discussion to cooling rates at temperatures below the equilibrium solidus. Between 1273 K (typically the lowest annealing temperature for 316LSS) and equilibrium solidus, the three points experience cooling rates between 1.15 × 10^4^ K/s and 2.91 × 10^4^ K/s; the latter rate is the highest rate encountered by any point. The lowest cooling rate experienced by any point is 1 K/s at 305 K. Meanwhile, the highest heating rate, 1.8 × 10^5^ K/s, is experienced by Y at 1074 K. The lowest heating rate between 1273 K and 1673 K experienced by Y is 2.08 × 10^3^ K/s at 1517 K.

The temperature v/s time curves shown in Fig. 7 are relevant for lamellae L1, L2 and L3. Meanwhile, lamella L4 should experience an initial cooling-heating-cooling similar to point X followed by 58 heating–cooling cycles at decreasing temperature rates. L5 should experience a single cooling cycle at a temperature rate lower than the maximum one shown in Fig. 7. Therefore, in the range of 300 K to 1673 K, any material point in the 3-layer and the 60-layer walls should experience cooling and heating rates from 1 K/s to 10^5^ K/s, which is typical for an LMD process^22^.

Now, we perform a thermodynamic analysis to understand why non-oxide precipitates are present in our LMD 316LSS walls. The first step involves analyzing the most stable (i.e., lowest Gibbs free energy) equilibrium phases that could exist in the liquid and solid states of 316LSS. This analysis is performed using the ThermoCalc software and the results are presented in section S4 of the supplementary material for several different temperatures in both the solid and liquid states. It reveals that the main phase in the solid 316LSS is γ-austenite and the main phase in the liquid is molten 316LSS, henceforth called Liquid A (LA). Amongst all the possible oxides, MnSiO_3_ is the most stable equilibrium phase in both the liquid and solid states; in the liquid state it exists in the form of Liquid B (LB), which is an Mn–Si–O rich liquid with composition close to MnSiO_3_ having trace amounts of Fe and S. Non-oxide (carbide, sulfide and phosphide) phases do not exist above the equilibrium liquidus temperature i.e., above 1703 K (1430 °C). Below the equilibrium solidus i.e., 1673 K (1400 °C), M–S (M = Mn, Cr and Fe in descending wt.%) is the most stable equilibrium non-oxide phase.

 Next, element solubility analysis is performed in order to understand the temperature dependent equilibrium composition and volume fraction of the most stable phases that coexist in the solid (γ-austenite, M-S and MnSiO_3_) and liquid (LA and LB) 316LSS.

**Figure 8:**
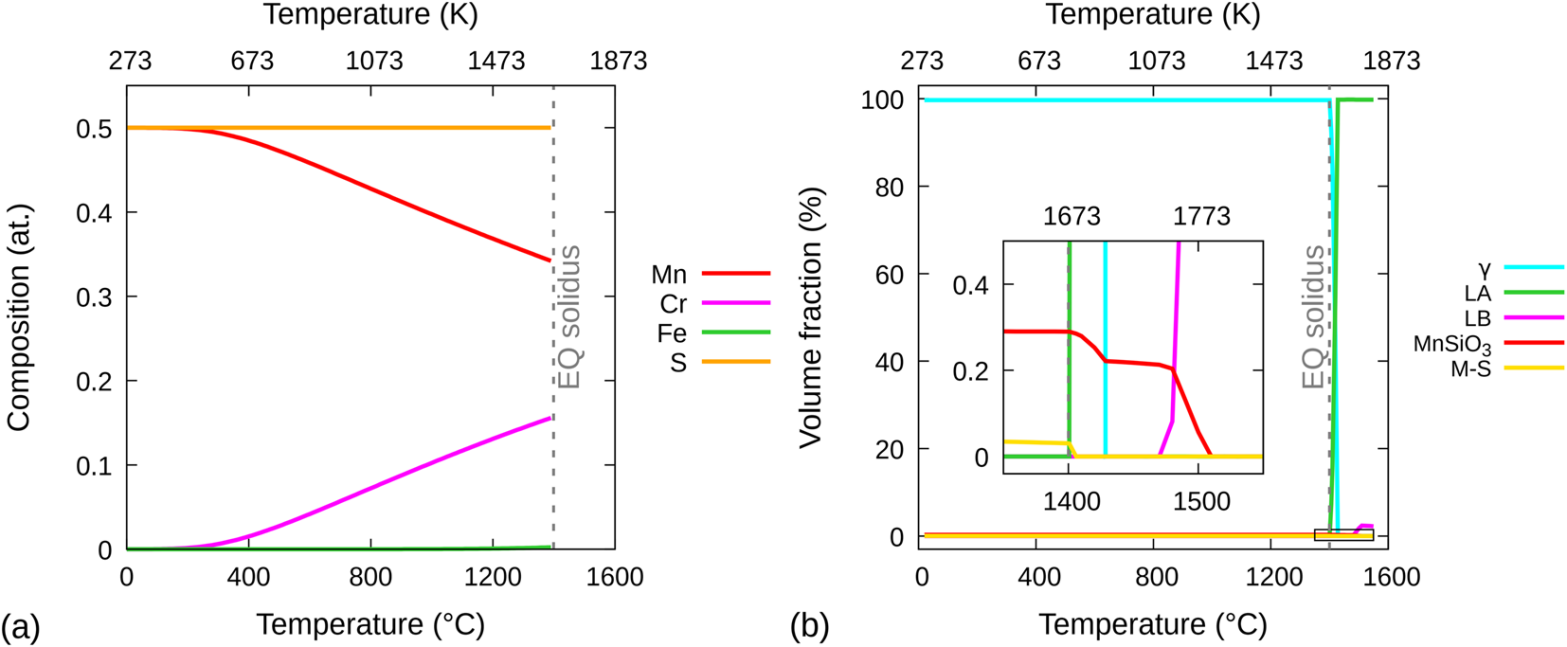
Equilibrium atomic composition and volume fraction curves. **(a)** Equilibrium atomic composition of only M–S (M = Mn, Cr and Fe), and **(b)** equilibrium volume fraction of γ-austenite, LA, LB, MnSiO_3_ and M–S, as a function of temperature. ‘EQ solidus’ stands for equilibrium solidus. Gnuplot v5.4 (http://www.gnuplot.info) was used to prepare this figure.

Figure 8a shows the equilibrium atomic composition and stoichiometry of M–S as a function of temperature. Starting as purely MnS below 473 K, the composition of M-S evolves between 473 K and equilibrium solidus as Mn is increasingly substituted mainly by Cr and in much lesser proportion by Fe from the γ-austenite. Meanwhile, the equilibrium atomic composition and stoichiometry of MnSiO_3_ (not shown) does not change with temperature until equilibrium solidus is reached.

Figure 8b shows the equilibrium volume fraction as a function of temperature for γ-austenite, M-S, MnSiO_3_, LA and LB. In the solid-state, γ-austenite is the dominant phase with some non-zero volume fraction of MnS and MnSiO_3_. Between equilibrium solidus (1673 K) and liquidus (1703 K), the phase fraction of γ-austenite decreases and that of LA correspondingly increases. At liquidus and beyond, all γ-austenite is replaced by LA. The volume fraction of MnSiO_3_ also decreases above 1673 K; however, up to 1703 K, the decrease in MnSiO_3_ is a combination of dissolution as well as the fact that the density of LA is lower than that of γ-austenite. At ~ 1743 K, MnSiO_3_ begins to transform into LB and the transformation is complete at ~ 1783 K. Meanwhile, just above equilibrium solidus, M-S dissolves into LA faster than γ-austenite transforms into LA; at 1683 K, all M-S has dissolved into LA.

The data used to plot the elemental solubility curves is then used to perform the precipitation kinetics simulations for nucleation and growth of M–S inclusions on MnSiO_3_. Recalling that TEM observations show 72.22% of the 36 mixed precipitates have oxide inclusions at their core and non-oxide inclusions surrounding the oxides, the most important assumption made during the precipitation kinetics simulations is that M-S inclusions can nucleate only on existing MnSiO_3_ inclusions (see Methods). The precipitation kinetics simulations are performed during solidification/cooling from the liquid phase and heating from the room temperature.

**Figure 9:**
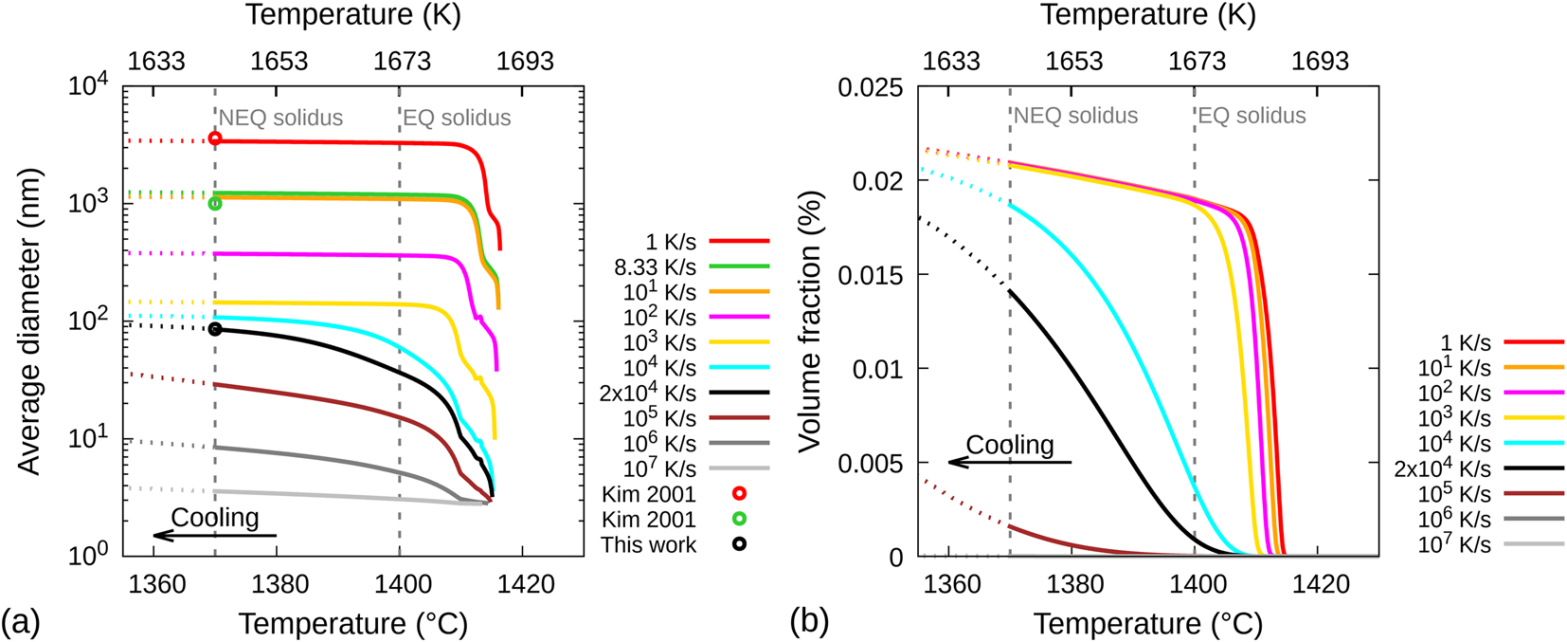
M–S precipitation kinetics simulation predicted **(a)** average diameter (log scale) and **(b)** volume fraction as a function of temperature during solidification/cooling at different temperature rates. ‘EQ solidus’ and ‘NEQ solidus’ respectively stand for the equilibrium and non-equilibrium solidus temperatures; the latter (1643 K) is obtained from Dépinoy et al.^30^ The black curves in **(a,b)** correspond to approximately the average cooling rate obtained from Fig. 7 between 1273 and 1673 K. The red and green empty circles in **(a)** represent the M–S precipitate diameters reported by Kim et al.^16^ for cooling rates 1 K/s and 8.33 K/s, respectively. The black empty circle represents the non-oxide inclusion diameter in mixed precipitates obtained from Fig. 6. Gnuplot v5.4 (http://www.gnuplot.info) was used to prepare this figure.

Figure 9a,b show the M–S diameter and volume fraction, respectively, as a function of temperature for different cooling rates, including 2 × 10^4^ K/s, which is approximately the average of the cooling rates encountered between 1273 K and 1673 K (Fig. 7). The non-equilibrium solidus 1643 K (1370 °C) reported by Dépinoy et al.^30^ is considered as the reference solidus temperature for these simulations. The model parameters have been chosen such that the predicted average diameter of M-S inclusions matches the measured average size of the non-oxide inclusions in the mixed precipitates in Fig. 6 for 2 × 10^4^ K/s at the non-equilibrium solidus. This fitting results in an excellent match between the predicted average diameters at cooling rates 1 K/s and 8.33 K/s and the experimental measurements of Kim et al.^16^; thus, validating not only the precipitation kinetics predictions but also FE predicted temperature rates.

For cooling rates 1 K/s, 10 K/s, 10^2^ K/s and 10^3^ K/s, precipitate growth saturates prior to reaching equilibrium solidus. For 10^4^ K/s, it saturates at the non-equilibrium solidus. For cooling rates 10^5^–10^7^ K/s, precipitates continue to grow below the non-equilibrium solidus, however, the increase in their diameter is overestimated below this temperature because the simulations have been performed using the diffusion coefficient of M–S in liquid (Methods). When the simulations are reperformed (not shown) using the diffusion coefficient of M–S in γ-austenite, which is approximately 5 orders of magnitude lower than that in liquid, the changes in diameters below the non-equilibrium solidus for all temperature rates are much smaller. Furthermore, at any cooling rate, M–S does not nucleate/grow below 1273 K i.e., below 0.76T_m_ (T_m_ = equilibrium melting point), which is also the lowest annealing temperature for 316LSS. The predicted average diameters of M–S at the non-equilibrium solidus are (in nm) 3395.1, 1131, 375.5, 144.5, 107.8, 29.1, 8.5 and 3.6, respectively, for cooling rates (in K/s) 1, 10, 10^2^, 10^3^, 10^4^, 10^5^, 10^6^ and 10^7^.

Figure 9b shows that the M–S volume fraction for cooling rates from 1 to 10^3^ K/s is ~ 0.021%. At cooling rates higher than 10^3^ K/s, the volume fraction begins to decrease faster than the increase in cooling rate. At 2 × 10^4^ K/s, the volume fraction is ~ 0.0145% at non-equilibrium solidus, which is still comparable to the one obtained at 1 K/s. At 10^5^ K/s, the M–S volume fraction is less than 0.002%, and it is negligibly small at 10^6^ and 10^7^ K/s. A significant change in M–S volume fraction is not expected for temperatures lower than non-equilibrium solidus at any cooling rate.

**Figure 10:**
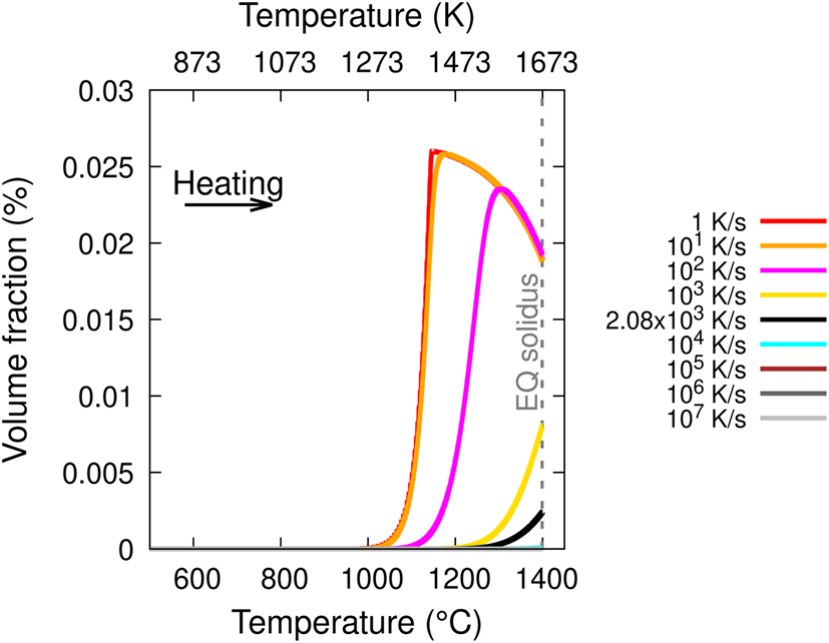
M–S precipitation kinetics simulation predicted volume fraction as a function of temperature during heating at different temperature rates. ‘EQ solidus’ stands for the equilibrium solidus temperature. The black curve corresponds to the minimum heating rate obtained from Fig. 7 between 1273 K and 1673 K. Gnuplot v5.4 (http://www.gnuplot.info) was used to prepare this figure.

Figure 10 shows the M–S volume fraction as a function of temperature for different heating rates, including 2.08 × 10^3^ K/s, which is the lowest predicted temperature rate encountered by point Y between 1273 K and 1673 K. For these simulations, the diffusion coefficient of M–S in γ-austenite has been used and, for simplicity, equilibrium solidus is considered as the reference solidus. Note that during rapid heating, the non-equilibrium solidus should be higher than the equilibrium solidus, however, this is not considered here because the precipitation kinetics simulations use the equilibrium solubility curve of M–S (Fig. 8) and this curve shows that M–S has already completely dissolved at only 10 K above the equilibrium solidus.

Similar to the solidification/cooling case, the volume fraction of M–S does not change below 1273 K at any heating rate. The volume fraction of M–S for heating rates 1 K/s and 10 K/s rapidly increases to 0.025% but only above 1273 K, and then decreases with increasing temperature until equilibrium solidus is reached. A similar trend occurs at 10^2^ K/s but starting from 1373 K and reaches a slightly lower maximum volume fraction than at 1 K/s. At 10^3^ K/s, the volume fraction starts to increase from 1473 K and reaches to ~ 0.008% at equilibrium solidus. At 2.08 × 10^3^ K/s, the volume fraction starts to increase from ~ 1573 K and reaches a maximum of 0.0025%, which is an order of magnitude lower than that occurring during cooling at 2 × 10^4^ K/s. For heating rates ≥ 10^4^ K/s, the change in M–S volume fraction is negligible at any temperature.

The most important results of the precipitation kinetics simulations are as follows: (i) there is no change to the M–S average diameter or volume fraction below 1273 K for any temperature rate, (ii) during solidification/cooling, the M–S volume fraction only increases at cooling rates lower than 10^5^ K/s in magnitude, and (iii) during heating, the maximum M–S volume fraction increases at rates lower than 10^4^ K/s.

Combining the results obtained from the FE simulations (Fig. 7) and the precipitation kinetics simulations, it can be seen that the M–S volume fraction increase during solidification/cooling at 1.15 × 10^4^ – 2.91 × 10^4^ K/s is higher by an order of magnitude than that during heating at the lowest rate 2.08 × 10^3^ K/s. Therefore, the nucleation and growth of M–S on oxide inclusions should be significant only during solidification/cooling at temperatures above 1273 K; the heating should play a negligible role. This explains why M-S inclusions have been observed in TEM lamellae L1–L5.

Since the inert gas atomization of 316LSS is expected to have similar maximum solidification/cooling rates as the LMD process, the precipitation kinetics simulations also explain why M–S inclusions in LMD 316LSS have a similar size to those observed in TEM lamella LP taken from the inert gas atomized 316LSS powder (Fig. 5).

Furthermore, the analysis also explains why M–S inclusions have not been observed in TEM studies of SLM 316LSS^2–4,7^. During SLM, the maximum temperature rates of 10^6^–10^7^ K/s can be encountered at temperatures above 1273 K^23^. At these rates, the predicted average diameters of M–S precipitates are very small and their volume fraction is negligible (Fig. 9), which makes it very difficult to detect them even with high-resolution STEM imaging.

Finally, although precipitation kinetics of non-oxide inclusions other than M–S have not been performed, nevertheless, similar thermodynamic arguments can also be used to explain their presence in LMD 316LSS and inert gas atomized 316LSS studied in this work, as well as their absence in SLM 316LSS in other works^2–4,7^.

In conclusion, the TEM studies performed in this work have revealed a plethora of different kinds of non-oxide inclusions (carbides, sulfides, phosphides and intermetallics) in the LMD 316LSS samples studied in this work. Furthermore, 70.9% of all the precipitates found in the TEM lamellae extracted from the LMD 316LSS samples are either purely non-oxide inclusions or mixed precipitates containing non-oxide inclusions together with oxide inclusions. These results prove that non-oxide inclusions can indeed form during DED-type AM processes such as LMD.

The presence/absence of non-oxide inclusions is dependent on the temperature rates experienced during processing as well as a minimum temperature below which they do not form. The precipitation kinetics simulations performed in this work show that new non-oxide inclusions form only during solidification/cooling (and not heating) at temperatures above 1273 K (1000 °C) i.e., 0.76T_m_, and at temperatures rates less than or equal to 10^5^ K/s. The FE simulations performed in this work, supported by existing literature^22^, reveal that the highest temperature rate magnitudes encountered during the LMD of 316LSS samples are less than 10^5^ K/s at temperatures above 1273 K; these thermal conditions occur only during the initial stages of the heating–cooling cycles in a material just after its deposition. Furthermore, similar rates must be experienced during inert gas atomization because the size of inclusions in gas atomized 316LSS are similar to the ones obtained in LMD 316LSS. These predictions explain the presence of non-oxide inclusions in both inert gas atomized 316LSS powder and LMD 316LSS samples. Meanwhile, the highest temperature rates typically encountered during powder-bed based techniques such as SLM at temperatures above 1273 K are in the range 10^6^–10^7^ K/s^23^. Therefore, non-oxide inclusions should not form during SLM 316LSS, which is fully supported by existing TEM studies^2–4,7^.

## Methods

### 316LSS powder properties and LMD process parameters

The 316LSS powder has been produced via the inert gas atomization process. This process involves slowly melting a wrought alloy and pouring it into an atomization chamber from the top. While being poured, the molten alloy is acted upon by high-speed inert gas (Ar) jets that disperse it into smaller droplets. These droplets rapidly solidify and cool during their descent and they are collected into a crucible at the bottom of the chamber. The solidification/cooling temperature rates attained during this process depend on several factors such as desired powder particle sizes, gas used, gas jet speed, etc. A sieve analysis has revealed the particle sizes range from 45 to 90 μm. Following the gas atomization process, the powder is typically stored in a sealed container, transported to the location where it is used for AM, and stored in a powder feeding chamber for the AM process.

The powder-based LMD process, also known as Direct Metal Deposition (DMD) or Laser Engineered Net Shaping (LENS), had been carried out inside the “Mobile” machine from BeAM. More details on the material, powder characteristics and specifications of the LMD machine can be found in ^31,32^.

A single-track bidirectionally-printed 3-layer wall had been built on a hot-rolled 316LSS substrate using this machine (Fig. 1a). A 3-layer wall ensures that (i) precipitates formed in the top layer are not affected by chemical heterogeneities on the substrate surface, and (ii) solidification/cooling rates are similar to those experienced by the first deposited layer during its deposition and comparable to those occurring during gas atomization. The LMD process parameters used to print the samples are summarized in Table 2. The final dimensions of the as-built wall are 100 mm × 0.8 mm × 0.6 mm.

**Table 2:** LMD process parameters used to build the 316LSS wall in Fig. 1.

Laser power	Powder flow rate	Deposition speed	Initial vertical position / vertical displacement of focusing head
225 W	0.1083 g/s	33.33 mm/s	3.5 mm above substrate / 0.2 mm after deposition of one layer

During the LMD process, the 316LSS powder particles are mixed with an inert gas (Ar in our case) and then transported to the focusing head, which contains laser fiber optic cable, gas and powder inlet, focusing lenses and a nozzle to direct the powder enveloped in inert gas. The laser melts the powder and the molten powder gets deposited on the substrate or the part being built. While the focusing head continues its trajectory to deposit the layer, the molten powder solidifies, typically in a few milliseconds, and then cools down, until the subsequent laser passage either on the same layer but adjacent to the material that is just deposited or on the layer above the deposited material. Subsequent deposition results in thermal cycling of the material in the solid-state and the number of cycles, their amplitudes and temperature rates depend on the number of laser passages and layers that remain to be built.

### Scanning Electron Microscopy (SEM): equipment and TEM lamellae preparation technique

The as-built wall and substrate had been mechanically cut near the mid-section and along the direction normal to the build and print directions (section B-B in Fig. 1a). This cross-section had been polished first using SiC papers with different grits (from 800-grit to 4000-grit), followed by diamond paste polishing with grit sizes 3 μm and 1 μm, and finally ion polishing. Then, this cross-section has been analyzed via SEM using a FEI Quanta 650 FEG Environmental-SEM microscope equipped with the symmetry detector (Oxford Instruments) for EBSD measurements.

The EBSD map shown in Fig. 1b for the 3-layer wall is a single map with a resolution of 2000 (height) × 1750 (width) pixels (1 × 0.88 mm^2^) of step size 0.5 μm. The acceleration voltage of the electron beam was 20 kV. The indexing success rate was 99%. The EBSD map shown in Fig. S1 for the 60-layer wall was stitched from 80 images of 1005 (width) × 692 (height) pixels (0.51 × 0.35 mm^2^) each with step size 0.5 μm. The acceleration voltage of the electron beam was 30 kV. The indexing success rate was 97.4%.

The 316LSS powder has also been analyzed via SEM. Some powder particles had been embedded inside a diluted conductive carbon cement and polished using the same approach as above.

Following the preliminary EBSD analysis, the sample had been transported to a FEI Helios Nanolab 660 dualbeam SEM microscope, which is equipped with a dual beam Focused Ion Beam (FIB). Using the standard lift-out process, the FIB-SEM is used to extract 4 TEM lamellae: 3 lamellae (L1, L2 and L3) from the 3-layer wall, 2 lamellae (L4 and L5) from the 60-layer wall, and 1 lamella (LP) from the powder sample.

## TEM: equipment and analysis

The six lamellae had been studied in an aberration-corrected FEI Titan^3^ G2 60–300 TEM microscope operating at 300 kV. This TEM is equipped with a Cs probe corrector, and a series of detectors including a High-Angle Annular Dark Field (HAADF) detector. It is also equipped with 4 EDS detectors, which allow generating chemical maps.

EDS takes advantage of the X-ray fluorescence to analyze the composition of the sample.

Working in STEM mode, it is possible to scan the sample and store the local X-ray spectrum at each pixel in the scanned zone. However, characteristic X-ray peaks from each element are convoluted with background contributions as well as any overlap with peaks of other elements; for example, the ${\mathrm{K}}_{\alpha }$ peak of S has an overlap with the ${\mathrm{L}}_{\alpha }$ peak of Mo. It is necessary to deconvolute these peaks in order to generate images separating the composition of each element in the scanned zone. The deconvolution procedure fits the experimental peaks using a linear combination of theoretical peaks of all the alloying elements in 316LSS. This method also removes the background contributions (e.g. Cu from the TEM grid). Following deconvolution, images are quantified using the standardless Cliff-Lorimer method^33^ to link experimental intensities to the relative amount of each element. All EDS images shown in this work are deconvoluted and quantified such that they are devoid of any thickness, density or peak overlap. EDS images of elements with lower concentration require brightness adjustment to facilitate visualization.

EDS line profile plots have been generated from the quantified EDS spectra for multiple precipitates in order to study the variation in elemental composition across different line paths through the precipitates. The plots have been generated by averaging over a width of 10 pixels.

Precipitate size (equivalent diameter) determination has been performed from the TEM images using the Fiji software.

## Heat transfer FE simulation setup

Solid-state heat transfer FE simulations have been performed to simulate LMD of the 3-layer wall in Fig. 1 in order to generate the temperature v/s time curves shown in Fig. 7.

### Governing equations and variational formulation

The heat transfer model used in this work is a continuum-based initial boundary value problem that accounts for heat conduction, convection and radiation based on the work of Weisz-Patrault^34^. Let $\Omega \left(t\right)\subset {\mathbb{R}}^{3}$ be a temporally evolving domain with boundary $\partial\Omega \left(t\right)$. Let $\mathcal{T}=\left[0,{t}_{max}\right]$ be the time interval of interest, where ${t}_{max}$ is known. The governing equations for the heat transfer problem at a material point ${\varvec{x}}\in\Omega (t)$ are:

1 $$\rho c_{v} \dot{T} = - \overline{\nabla } \cdot \overline{q}\quad {\text{in}}\quad {\Omega }\left( {\text{t}} \right) \times {\mathcal{T}}$$

2 $$\overline{q} = - \overline{\overline{K}} \cdot \overline{\nabla T}\quad {\text{in}}\quad {\Omega }\left( {\text{t}} \right) \times {\mathcal{T}}$$

The initial conditions are:

3 $$T\left( {x,0} \right) = T_{ini} \quad \forall x \in {\Omega }\left( 0 \right)$$

The boundary conditions are:

4 $$T\left( {x,t} \right) = T_{0} \quad {\text{on}}\quad \partial {\Omega }_{base} \times {\mathcal{T}}$$

5 $$q_{laser} \left( {x,t} \right) = 2\eta_{laser} \frac{{P_{laser} }}{{\pi R_{laser}^{2} }}\exp \left( { - \frac{{2\parallel x - x_{laser} \parallel^{2} }}{{R_{laser}^{2} }}} \right)\quad {\text{on}}\quad \partial {\Omega }_{laser} \left( t \right)$$

6 $$q_{Ar} \left( {x,t} \right) = h_{Ar} \left( {T - T_{inf} } \right)\exp \left( { - \frac{{2\parallel x - x_{laser} \parallel^{2} }}{{R_{Ar}^{2} }} } \right) \quad {\text{on}}\quad \partial {\Omega }_{Ar} \left( t \right)$$

7 $$q_{air} \left( {x,t} \right) = h_{air} \left( {T - T_{inf} } \right) \quad {\text{on}}\quad \partial {\Omega }_{air} \left( t \right)$$

8 $$q_{rad} \left( {x,t} \right) = \sigma {\varepsilon }\left( {T^{4} - T_{inf}^{4} } \right) \quad {\text{on}}\quad \partial {\Omega }\left( t \right)$$

where $T$ is the absolute temperature, $\overline{\overline{K}}$ is the second order thermal conductivity tensor, ${c}_{v}$ is the specific heat at constant volume, $\overline{\nabla }$ is the differential operator vector, $\overline{q}$ is the heat flux vector, $\overline{\nabla \theta }$ is the temperature gradient, ${T}_{ini}$ is the initial temperature $\partial {\Omega }_{base}$ is the bottom surface of the baseplate that does not evolve with time and it is applied a Dirichlet boundary condition. $\partial {\Omega }_{laser}\left(t\right)$, $\partial {\Omega }_{Ar}\left(t\right)$, $\partial {\Omega }_{air}\left(t\right)$ and $\partial {\Omega }_{rad}(t)$ are defined as those surfaces that have been respectively subjected to surface heat flux from the laser beam ${q}_{laser}$, forced convective heat transfer ${q}_{Ar}$ due to the Ar-gas flowing from the nozzle of the focusing head, natural air convective heat transfer ${q}_{air}$ and radiative heat flux ${q}_{rad}$, respectively; note that these surfaces evolve with time. ${h}_{Ar}$ and ${h}_{air}$ are the convective heat transfer coefficients for Ar-gas and air, respectively. $\sigma$ and $\epsilon$ are the Stefan-Boltzmann constant and emissivity of the deposited material, respectively. ${T}_{inf}$ is the infinitive temperature. The laser beam interaction with the material is modeled as a surface heat flux condition having a 2D Gaussian distribution with maximum power ${P}_{laser}$ and absorptivity ${\eta }_{laser}$ acting on a circular area with radius ${R}_{laser}$ centered at ${x}_{laser}$. The Ar-gas heat flux is also modeled as a Gaussian with its peak at the center of the laser beam ${x}_{laser}$ and a radius ${R}_{Ar}>{R}_{laser}$ that is large enough to cover some area around the build.

At a given time step, the following variational (weak) form of the governing equations with the boundary conditions is solved:

9 $${\int }_{\Omega }\rho {c}_{\varepsilon }\frac{\left({T}_{n}^{h}-{T}_{n-1}^{h}\right)}{\Delta t}\widehat{T} d\mathrm{V}={\int }_{\Omega }{\overline{q} }^{h}\cdot \overline{\nabla \widehat{T}}\mathrm{d }V -{\int }_{\partial {\Omega }_{\mathrm{laser}}}{q}_{laser}\widehat{T}\mathrm{dS}-{\int }_{\partial {\Omega }_{\mathrm{Ar}}}{q}_{Ar}\widehat{T}\mathrm{dS}-{\int }_{\partial {\Omega }_{\mathrm{air}}}{q}_{air}\widehat{T}\mathrm{dS}-{\int }_{\partial {\Omega }_{\mathrm{rad}}}{q}_{rad}\widehat{T}\mathrm{dS}$$

where a Euler backward time integration scheme has been used, $\widehat{T}$ is the temperature test function, ${T}^{h}$ is the approximate solution sought, ${\overline{q} }^{h}=-\overline{\overline{K}}\cdot {\overline{\nabla T} }^{h}$ and the subscript $n$ is the current time step.

The weak form has been implemented in the FEniCS^35^ open-source (LGPLv3) FE computational platform for solving partial differential equations and the non-linear Newton iterative solver is used.

### Mesh

The simulated geometry shown is created based on the experimental setup (Fig. 1). It consists of a baseplate (not shown in Fig. 1 but present during the experiment), the hot-rolled 316LSS substrate, and the 3-layer LMD 316LSS wall. The baseplate, substrate and wall are created as independent volumes and merged to form a single material volume for the simulations. The geometry is then decomposed into subdomains as shown in Fig. S5 and meshed using the open-source gmsh^36^ software.

The structured regions of the substrate and baseplate were meshed using 6 mm seeds and the structured region of wall was meshed using 0.1 mm seeds. An unstructured region was necessary to transition from the 0.1 mm seeds of the wall to the 6 mm seeds of the substrate and baseplate. This unstructured region starts around the wall and goes to a distance of 15 mm in all directions to ensure a uniform increase in mesh element size. All elements are chosen to be tetrahedral with quadratic interpolation (P2). A conformal mesh is obtained after recombining using “transfinite” mesh options in gmsh (Fig. S6).

For simplicity, mass addition is simulated via a mesh element addition procedure. The initial mesh only contains the substrate and the baseplate. When the laser passes over the region where the first mass is added, a new element is generated at the location where the laser passes. However, this element is not added to the existing mesh. Instead, a new geometry and mesh is generated. This new mesh is an augmentation of the geometry and mesh of the previous time step with one additional geometric element to mimic the deposition of some material on a layer. At the beginning of the deposition of each layer, a cuboid element of size 0.6 mm (along $x$) × 0.6 mm (along $y$) × 0.2667 mm (along $z$) is generated. Each of the subsequent elements added to that layer are of size 0.1 mm × 0.6 mm × 0.2667 mm. Finally, for the 3-layer simulation, the initial and final FE meshes are composed of 170056 and 457948 elements, respectively, with approximately 100 mesh elements added for the additional geometry element.

### Simulation setup

The FE simulation parameters are presented in Table 3. The baseplate has the same length and breadth as the substrate but its thickness is 40 mm (5 times the thickness of the substrate in Fig. 1). At the first timestep, a cuboidal element of 0.6 mm × 0.6 mm × 0.2667 mm is generated and initialized to 1885 K. The laser beam and the Ar-gas jet are centered on the top surface of this element and although the element is initialized to 1885 K, the action of the Ar-gas almost instantaneously reduces the temperature to ~ 1773 K (1500 °C), which is similar to the melt temperature at deposition. All the remaining surfaces are provided with natural air flow ${q}_{air}$ boundary condition. In addition, radiation heat loss condition ${q}_{rad}$ is imposed over all surfaces $(\partial {\Omega }_{rad}=\partial\Omega$).

**Table 3:** Parameters for the FE simulations appearing in Eqs. (1)–(9).

$\sigma$(W m^−2^ K^−4^)	5.67 × 10^–8^
$\varepsilon$	0.65
${c}_{\varepsilon }$(J kg^−1^ K^−1^)	500
$\eta$	0.9
${h}_{Ar}$(W m^−2^ K)	12,500 × 10^6^
${h}_{air}$(W m^−2^ K)	15 × 10^6^
${T}_{ini}={T}_{0}={T}_{sub}$(K)	300
${R}_{Ar}$(mm)	20
${R}_{laser}$(mm)	0.6
${K}_{11}={K}_{22}={K}_{33}$(W mm^−1^ K^−1^)	16.3
$\rho$(kg m^−3^)	8000
${P}_{laser}$(W)	225

The simulation starts with the first deposition at $t=0$ s. The simulation time step is 3 ms. Deposition of each layer takes 994 steps i.e., 2.982 s; this value corresponds to a deposition speed of 33.53 mm/s, which is very close to LMD deposition speed of 33.33 mm/s. Between successive layer depositions, a dwell time of 1.221 s (407 steps) is provided to account for focusing head deceleration and reverse acceleration. After the deposition of the final layer, a dwell time of 0.609 s (204 steps) is provided. Therefore, the total simulation steps are 4000, which correspond to a total simulated time of 12 s. The simulation was performed on a single CPU thread and it took 22 h 26 min 12 s to complete.

## Thermodynamic calculations

The phase stability and elemental solubility analysis is performed using the Thermo-Calc software version 2019b with the TCFE9 database.

A mean field precipitate kinetics model based on the classical nucleation and growth theories^37–41^ is used to predict the nucleation, growth and coarsening kinetics of M–S precipitates on MnSiO_3_ precipitates already present in γ-austenite/LA. M–S precipitates are assumed to be spherical and their growth is assumed to be controlled by the diffusion in γ-austenite/LA. Their growth rate is calculated following Zener’s approximate solution for spherical precipitates^42^. The Gibbs–Thomson effect^43^ is also taken into account. γ-austenite/LA is assumed to be an ideal supersaturated solid/liquid solution containing both interstitial and substitutional elements. The solubility of each element in γ-austenite, MnSiO_3_, M-S, LA and LB is taken from the ThermoCalc software generated data used to plot Fig. 8.

An important step in the precipitation kinetics simulations is to estimate the number of potential nucleation sites for M-S. In order to understand how these sites are chosen, the TEM results for L1 – L5 are reconsulted (Fig. 3, S3 and S6). These results show that 72.22% of all the mixed precipitates contain oxide inclusions at their core and non-oxide inclusions surround them. These numbers strongly suggest that non-oxide inclusions mainly nucleate on oxide inclusions, a phenomenon that has already been observed in other studies performed at slower cooling rates^15,16^. Meanwhile, Fig. 6 shows that (i) the average size of oxide precipitates is larger than the average size of oxide inclusions in mixed precipitates and (ii) with the exception of 2 oxide inclusions in mixed precipitates, the remaining oxide inclusions are either in the size range of oxide precipitates or smaller than the smallest oxide precipitate. These results show that the growth of non-oxide inclusions could stunt further growth of oxide inclusions. Therefore, when computing the number of potential nucleation sites for M–S, the following assumptions have been made: (i) only one M–S inclusion grows on one oxide inclusion, and (ii) the diameter of an oxide inclusion is the average size of the oxide inclusions in mixed precipitates from Fig. 6 i.e., 116.19 nm.

Oxide inclusions are assumed to be only MnSiO_3_ and to have a spherical shape. We also assume that the maximum possible equilibrium phase fraction of MnSiO_3_ (Fig. 8) is present in γ-austenite/LA. The composition of γ-austenite/LA is assumed to be such that all oxygen is used up to form MnSiO_3_; it is as follows (in at. %): Cr—18.063, Ni—12.027, Mo—1.448, Mn—1.464, Si—1.331, P—0.025, S—0.009, C—0.05 and Fe—balance. In the following, a maximum volume fraction of ${f}_{max}^{o}=0.3\%$ (rounded up from the equilibrium calculation below solidus in Fig. 8) is considered for MnSiO_3_.

The diameter of MnSiO_3_ precipitates is taken as the average diameter of oxide inclusions in mixed precipitates from Fig. 6. From the average mixed oxide volume (${V}_{r}^{o}=\frac{4}{3}\pi {r}^{3}$ with $r=58.095\times {10}^{-9}$ m from Fig. 6) and ${f}_{max}^{o}$, the MnSiO_3_ number density (${N}^{o}$ m^-3^) can be estimated as:

$${N}^{o}=\frac{{f}_{max}^{o}}{{V}_{r}^{o}}=\frac{0.003}{8.213\times {10}^{-22}}=3.65\times {10}^{18}/{m}^{3}$$

We have assumed that only one M–S precipitate nucleates on one MnSiO_3_. Therefore, ${N}^{o}$ is the potential number of nucleation sites for M–S nucleation on MnSiO_3_. Finally, the diffusion coefficient is given as:

$$D=\alpha {D}_{0}\mathrm{exp}\left(-\frac{{Q}_{0}}{RT}\right),$$

where ${D}_{0}$ (m^2^/s) is the pre-exponent factor, ${Q}_{0}$ (J/mol) is the activation energy, $R$ is the universal gas constant, $T$ is the temperature, and $\alpha$ is a fitting parameter.

The parameters used for the precipitate kinetics simulations are reported in Table 4; some were either obtained from the literature^41,44^ or fitted to reproduce the measured non-oxide inclusion size reported in Fig. 6. For further information about this well documented model see^38,40,45^. The model calculates a particle size distribution following the Euler-type multi-class approach^38^ and only the average diameter of the particle is plotted in Fig. 9a.

**Table 4:** Precipitation kinetics model parameters^41,44^.

Molar volume of γ-austenite (m^3^/mol)	7 × 10^–6^
Molar volume of M-S (m^3^/mol)	1.1 × 10^–5^
Interfacial energy (J/m^2^)	0.25
Heterogeneous nucleation factor	0.5
Mn in γ-austenite	D_0_ = 0.178 × 10^–4^, Q_0_ = 2.64 × 10^5^, α = 1
Mn in LA	D_0_ = 1.8 × 10^–7^, Q_0_ = 1.3 × 10^4^, α = 1
Cr in γ-austenite	D_0_ = 0.169 × 10^–4^, Q_0_ = 2.639 × 10^5^, α = 1
Cr in LA: D (m^2^/s)	D = 4.9 × 10^–9^
S in γ-austenite	D_0_ = 7.52 × 10^–4^, Q_0_ = 2.364 × 10^5^, α = 1
S in LA	D_0_ = 4.33 × 10^–8^, Q_0_ = 3.5 × 10^4^, α = 1.45

## Supplementary Information

Supplementary Information.
