# Supplementary material for: Non-oxide precipitates in additively manufactured austenitic stainless steel
Source: Sci Rep. 2021 May 17;11:10393. doi: 10.1038/s41598-021-89873-2 (PMC8128905; doi:10.1038/s41598-021-89873-2)
Supplement: Supplementary file 1 — Supplementary Information. [file 41598_2021_89873_MOESM1_ESM.pdf]

Non-oxide precipitates in additively manufactured austenitic stainless steel

Manas V. Upadhyay[a,*], Meriem Ben Haj Slama[a,b], Steve Gaudez[a,c], Nikhil Mohanan[a], Lluis Yedra[b,d,1], Simon Hallais[a], Eva Héripré[b], Alexandre Tanguy[a]

[a]*Laboratoire de Mécanique des Solides (LMS), CNRS UMR 7649, Ecole Polytechnique, Institut Polytechnique de Paris, 91128 Palaiseau, France*

[b]*Laboratoire de Mécanique des Sols, Structures et Matériaux (MSSMat), CNRS UMR 8579, CentraleSupélec, Université Paris-Saclay, 91190 Gif-sur-Yvette, France*

[c]*Institut Jean Lamour (IJL), CNRS UMR 7198, Université de Lorraine, Campus ARTEM Nancy, France*

[d]*Laboratoire Structures, Propriétés et Modélisation des Solides (SPMS), CNRS UMR 8580, CentraleSupélec, Université Paris Saclay, 91190 Gif-sur-Yvette, France*

*Corresponding author. Email: manas.upadhyay@polytechnique.edu. Tel: +33 (0)1 69 33 58 12

Abstract: Precipitates in an austenitic stainless steel fabricated via any Additive Manufacturing (AM), or 3D printing, technique have been widely reported to be only Mn-Si-rich oxides. However, via Transmission Electron Microscopy (TEM) studies on a 316L stainless steel, we show that non-oxide precipitates (intermetallics, sulfides, phosphides and carbides) can also form when the steel is fabricated via Laser Metal Deposition (LMD) – a directed energy deposition-type AM technique. An investigation into their origin is conducted with support from precipitation kinetics and finite element heat transfer simulations. It reveals that non-oxide precipitates form during solidification/cooling at temperatures $\geq 0.75T_m$ (melting point) and temperature rates $\leq 10^5$ K/s, which is the upper end of the maximum rates encountered during LMD but lower than those encountered during Selective Laser Melting (SLM) – a powder-bed type AM technique. Consequently, non-oxide precipitates should form during LMD, as reported in this work, but not during SLM, in consistency with existing literature.

Keywords: transmission electron microscopy, directed energy deposition, gas-atomization, laser metal deposition, precipitate

[1] Current address: University of Barcelona, Department of Electronics and Biomedical Engineering and Institute of Nanoscience and Nanotechnology (IN2UB), 08028 Barcelona, Catalonia, Spain

Supplementary Data

S1. SEM and TEM analysis of precipitates in lamellae L4 and L5

In addition to the 3-layered wall studied in the main article, a 60-layered wall was printed using the same parameters as those shown in table 2. The final dimensions of the wall were 100 mm (length) x 11.85 mm (height) x 0.6 mm (width). This 60-layered wall was cut at its mid length and the cross-section was polished for EBSD analysis. The EBSD map of the cross-section of this 60-layered wall is shown in Fig. S1 below.

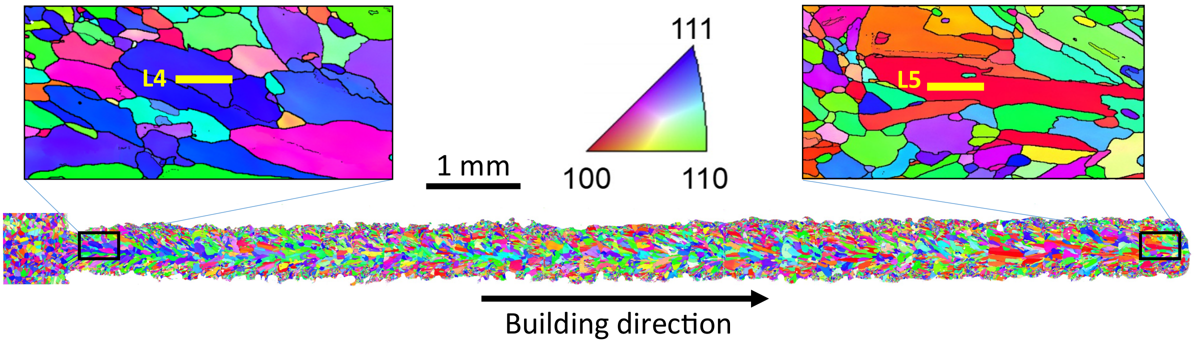

Figure S1. EBSD orientation map of the 60-layer LMD 316LSS wall of Fig. 1a. *The map is color-coded according to IPF along building direction at a cross-section approximately at the mid-section of the 60-layer wall. Subfigures in show zoomed-in EBSD maps. Yellow-colored lines demark zones underneath which lamellae L4 and L5 are extracted (in the out-of-plane direction). These lines correspond to the top side of L4 and L5 in the HAADF images in Fig. S2. Aztec v4.2 (Oxford Instruments https://nano.oxinst.com/products/aztec/) and Microsoft PowerPoint have been used to prepare this figure.*

Two TEM lamellae, L4 and L5, were extracted from the zones highlighted in Fig. S1 and characterized in the Titan^3 TEM. The EBSD parameters used to generate Fig. S1 are given in the methods section. Fig. S2 shows the HAADF and EDS maps of L4 and L5 and Fig. S3 shows the HAADF and EDS maps of each non-oxide precipitate in L4 and L5.

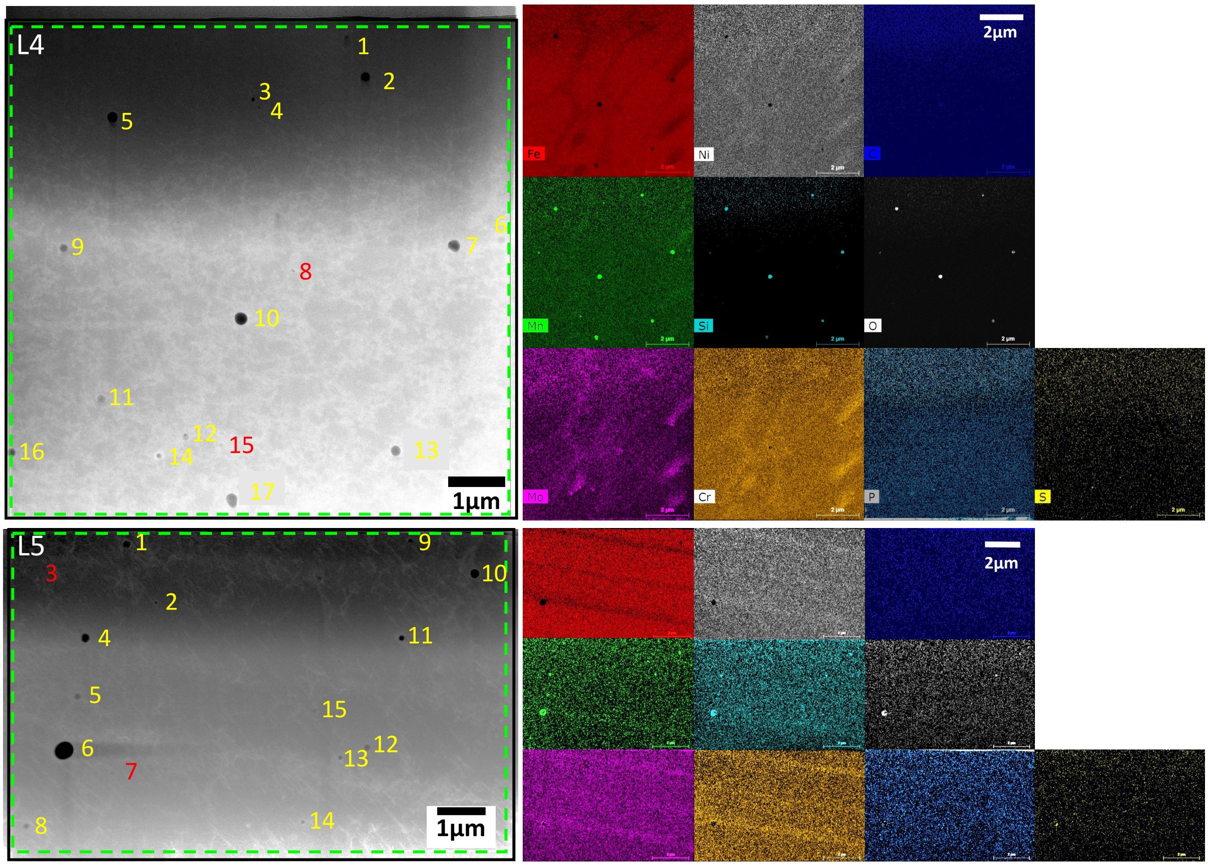

***Figure S2**: **STEM HAADF images and EDS chemical maps of L4 and L5 extracted from the 60-layer wall of Fig. S1.** EDS maps have been generated from within the black outlined region in each HAADF image. The color scheme for the EDS maps is the same as the one used in Fig. 2. The dotted green lines in each HAADF image represents the zone where the lamella composition has been computed. Precipitates that are easily visible in the HAADF images are numbered in yellow font. Precipitates that have been detected after high resolution imaging are numbered in red font. Scale for EDS maps of each lamella are shown in their C map. HAADF images and EDS maps have been acquired using the TIA v4.6 (FEI www.fei.com) and the Esprit v1.9 (Brucker https://www.bruker.com), respectively. Fiji v2.1.0/1.53c (https://fiji.sc/) and Microsoft Powerpoint have been used to prepare this figure.*

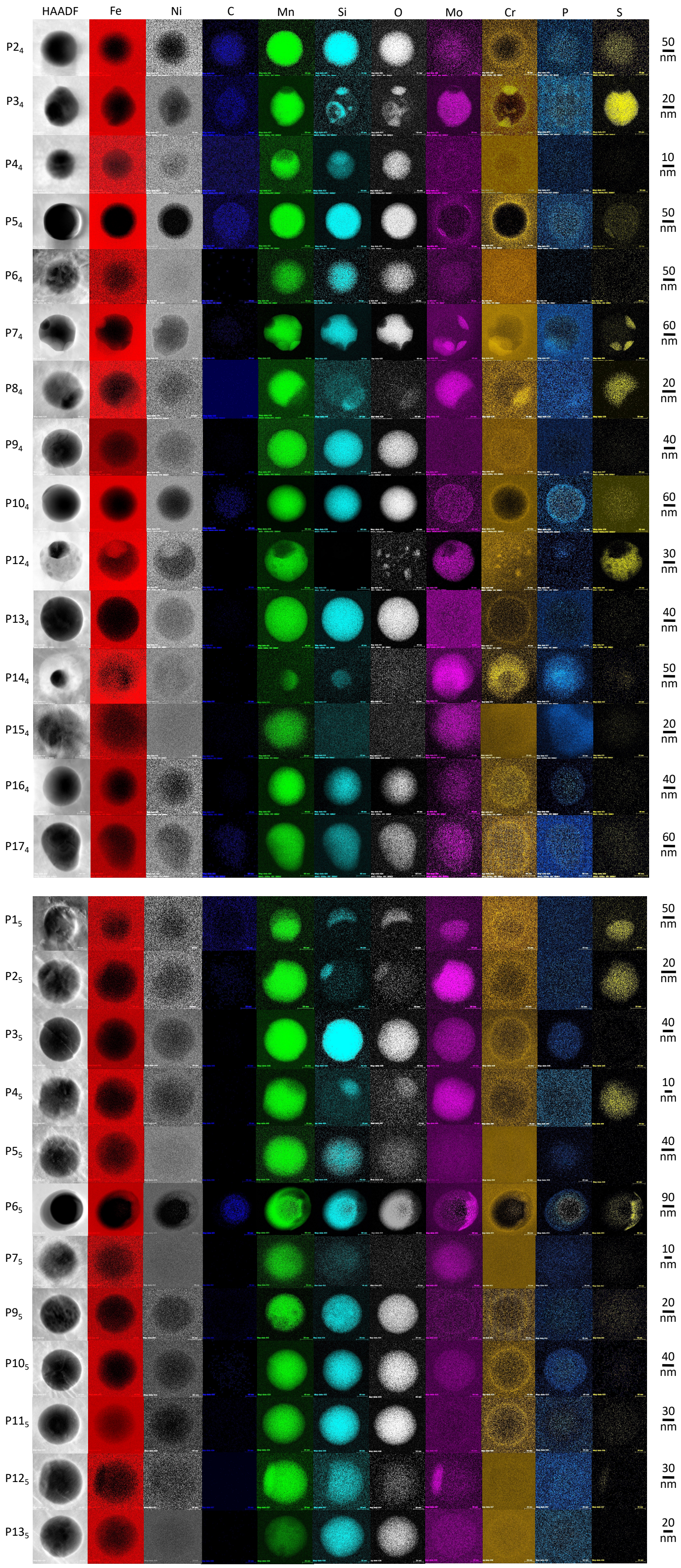
HAADF
Fe
Ni
C
Mn
Si
O
Mo
Cr
P
S
$P2_4$
$P3_4$
$P4_4$
$P5_4$
$P6_4$
$P7_4$
$P8_4$
$P9_4$
$P10_4$
$P12_4$
$P13_4$
$P14_4$
$P15_4$
$P16_4$
$P17_4$
$P1_5$
$P2_5$
$P3_5$
$P4_5$
$P5_5$
$P6_5$
$P7_5$
$P9_5$
$P10_5$
$P11_5$
$P12_5$
$P13_5$
50 nm
20 nm
10 nm
50 nm
50 nm
60 nm
20 nm
40 nm
60 nm
30 nm
40 nm
50 nm
20 nm
40 nm
60 nm
50 nm
20 nm
40 nm
10 nm
40 nm
90 nm
10 nm
20 nm
40 nm
30 nm
30 nm
20 nm

Figure S3: STEM HAADF images and EDS maps of all non-oxide and mixed precipitates in L4 and L5. *To facilitate visualization, picture corrections have been made for all the EDS maps. HAADF images and EDS maps have been acquired using the TIA v4.6 (FEI www.fei.com) and the Esprit v1.9 (Brucker https://www.bruker.com) software, respectively. ImageJ and Microsoft PowerPoint have been used to prepare this image. Fiji v2.1.0/1.53c (https://fiji.sc/) and Microsoft Powerpoint have been used to prepare this figure.*

S2. Chemical composition of precipitates in L1 – L3

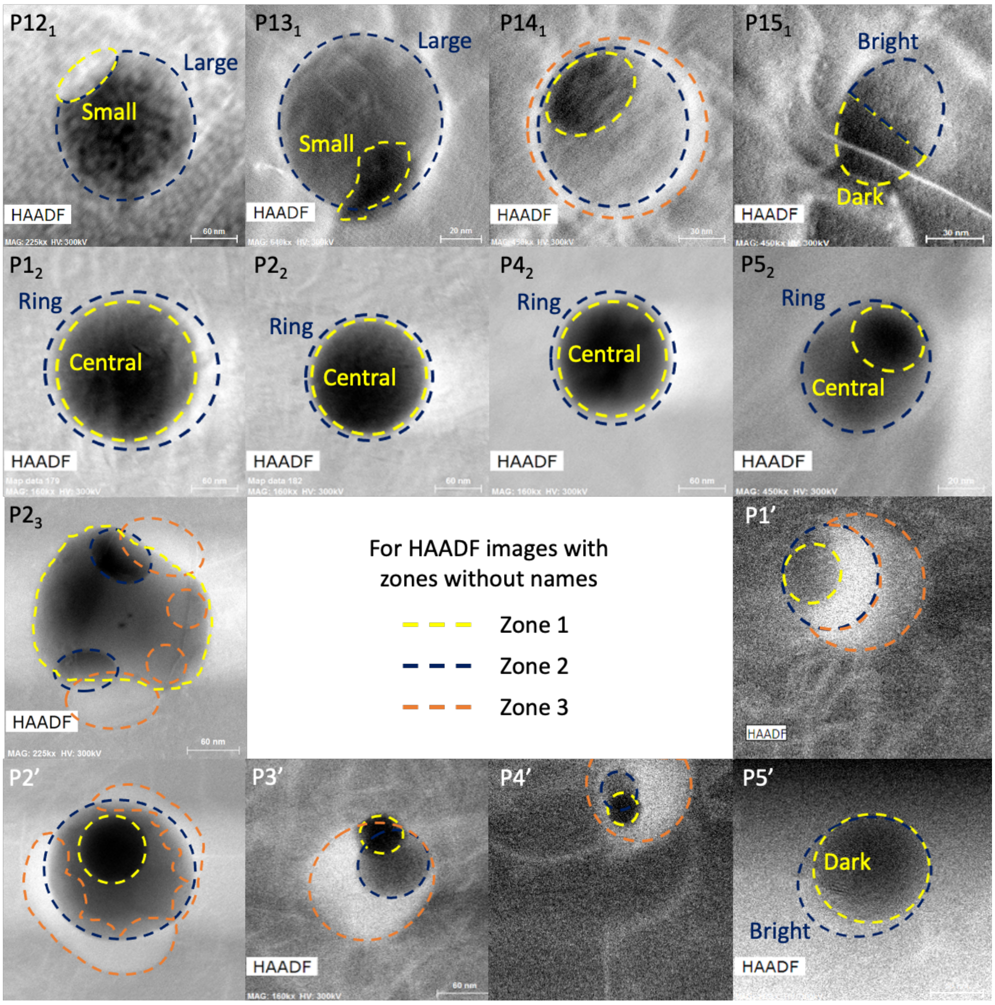

Figure S4: Nomenclature of regions in mixed precipitates in L1, L2, L3. *For the names of the precipitates, refer to Fig. 3 and 5. HAADF images have been acquired using the TIA v4.6 (FEI www.fei.com) software. Fiji v2.1.0/1.53c (https://fiji.sc), gnuplot v5.4 (http://www.gnuplot.info) and Microsoft PowerPoint have been used to prepare this image.*

Table S1: Elemental concentrations in L1, L2, L3, L4, L5 and LP obtained from the quantification table of EDS maps in Fig. 2 and S2, and the concentrations of precipitates/inclusions in L1, L2, L3 and LP measured from EDS line profiles maps in comparison with the wrought 316LSS composition. *Refer to Fig. 3, 4 and S4 for precipitate/inclusion nomenclature. For precipitates/inclusions, elements are presented in colored font. Red and blue fonts are used for elements whose compositions are higher*

and lower, respectively, than their composition in the wrought alloy. Elements highlighted in yellow are those whose composition in the precipitate/inclusion is higher than their composition in the wrought alloy but lower than their composition in the lamella to which the precipitate belongs. Elements highlighted in cyan are those whose composition in the precipitate/inclusion is lower than their composition in the wrought alloy but higher than their composition in the lamella to which the precipitate belongs.

	Fe	Cr	Ni	Mo	Mn	Si	O	P	S	C
Wrought	65.6	16.9	12.7	2.5	1.5	0.7	0.0	0.015	0.005	0.011
L1	61.59	14.77	13.24	2.24	2.26	1.35	1.08	0.36	0.16	2.94
L2	59.74	13.56	14.02	2.31	2.19	1.73	1.11	0.4	0.09	4.86
L3	54.53	13.89	12.52	3.35	1.96	1.7	3.69	0.7	0.41	7.25
L4	64.13	14.07	15.59	2.07	2.23	0.51	0.44	0.27	0.07	0.62
L5	63.16	13.6	16.33	2.56	2.37	0.53	0.53	0.28	0.1	0.54
LP	59.95	14.71	12.73	3.43	2.21	0.82	1.89	0.46	0.3	3.49

Precipitates		Higher than wrought (max. wt. %)	Lower than wrought (min. wt. %)
$P1_1$	Zone 1	Mo (5.5) Mn (10) Si (9) O (9) P (0.9) S (44) C (5)	Fe (4) Cr (10) Ni (0.5)
	Zone 2	Cr (21) Mo (11.7) Mn (4.4) Si (1.8) O (4.5) P (2.7) S (20) C (3)	Fe (30) Ni (5)
	Zone 3	Cr (24) Mo (5.5) Mn (3.3) Si (2.1) O (2.5) P (2) S (2.5) C (2)	Fe (45) Ni (8.5)
	Smaller ellipses 1	Mo (12) Mn (3.8) Si (7) O (13.5) P (1.2) S (21) C (5.4)	Fe (20) Cr (12) Ni (3.7)
	Smaller ellipses 2	Mo (11) Mn (11) Si (7) O (14) P (1.7) S (16) C (5)	Fe (16.7) Cr (15) Ni (2.9)
	Smaller ellipses 3	Cr (24) Mo (7.7) Mn (4) Si (2) O (4) P (3.4) S (5.7) C (2.3)	Fe (40) Ni (7)
	Smaller ellipses 4	Cr (17.5) Mo (10) Mn (8.7) Si (3.5) O (11.3) P (1.7) S (14.7) C (5)	Fe (27) Ni (4.5)
	Smaller ellipses 5	Mo (9.4) Mn (5.5) Si (3) O (13.5) P (1.5) S (15) C (3.75)	Fe (32) Cr (16.2) Ni (6)
$P2_1$		Mn (13) Si (3.3) O (4.8) P (0.44) S (0.27) C (2.9)	Fe (47) Cr (16.4) Ni (9.5) Mo (2)
$P3_1$		Mn (5.3) Si (2.2) O (3.8) P (0.4) S (0.23) C (2.3)	Fe (58) Cr (15.7) Ni (10.7) Mo (1.9)
$P4_1$		Mn (8.3) Si (1.5) O (1.3) P (0.3) S (0.18) C (3.3)	Fe (54) Cr (15.6) Ni (11.2) Mo (1.9)
$P5_1$		Mn (5.3) Si (1.2) O (1) P (0.3) S (0.38) C (2.5)	Fe (59) Cr (15.6) Ni (12) Mo (2.1)
$P6_1$		Mn (8) Si (2.8) O (5.4) P (0.4) S (0.33) C (2.7)	Fe (55) Cr (16.1) Ni (8.8) Mo (1.65)
$P7_1$		Mn (4.6) Si (1.7) O (2.6) P (0.3) S (0.11) C (1.7)	Fe (59) Cr (14.5) Ni (12) Mo (1.55)
$P8_1$		Mn (5.9) Si (1.2) O (0.9) P (0.32) S (0.18) C (2)	Fe (59) Cr (16) Ni (11) Mo (1.7)
$P9_1$	Zone 1	Cr (18) Mo (3.7) Mn (15) Si (2.4) O (2.4) P (0.8) S (3.5) C (1.3)	Fe (42) Ni (7.7)
	Zone 2	Cr (17.6) Mo (4.2) Mn (20.8) Si (2.8) O (2.8) P (0.7) S (4) C (1)	Fe (40.3), Ni (7.7)
	Zone 3	Cr (26) Mo (5) Mn (5.8) Si (2) O (1.6) P (1.4) S (3.5) C (1.3)	Fe (47), Ni (7.6)
$P10_1$		Mn (6.1) Si (2) O (2.3) P (0.4) S (0.22) C (2.8)	Fe (57) Cr (15.2) Ni (10.7) Mo (2.1)
$P11_1$		Mo (3.3) Mn (3.5) Si (1.8) O (2.1) P (0.6) S (0.4) C (2.8)	Fe (55) Cr (15.7) Ni (11.8)
$P12_1$	Large	Mo (4) Mn (8.4) Si (2.3) O (3.5) P (0.66) S (0.7) C (5.8)	Fe (48) Cr (15.5) Ni (11.6)
	Small	Mo (3.1) Mn (2.4) Si (1.5) O (1.7) P (0.5) S (0.4) C (4.2)	Fe (54) Cr (15.5) Ni (13)
$P13_1$	Large	Mo (2.8) Mn (3.7) Si (1) O (1.1) P (0.43) S (0.3) C (3.8)	Fe (59) Cr (15.1) Ni (12.5)
	Small	Mo (3.5) Mn (6.4) Si (1.1) O (1.2) P (0.43) S (0.6) C (3.6)	Fe (56) Cr (14.6) Ni (11.9)
$P14_1$	Zone 1	Mo (2.3) Mn (3.5) Si (0.95) O (0.68) P (0.4) S (0.36) C (2.8)	Fe (57.8) Cr (15.7) Ni (11.6)
	Zone 2	Mo (3.3) Mn (8.5) Si (0.96) O (0.68) P (0.4) S (0.45) C (2.4)	Fe (56.5) Cr (15.3) Ni (11.2)
	Zone 3	Mo (2.6) Mn (5.6) Si (0.95) O (0.59) P (0.5) S (0.27) C (2.8)	Fe (60) Cr (16.4) Ni (12.1)
$P15_1$	Dark	Ni (13.11) Mo (3.4) Mn (5.2) Si (1) O (0.75) P (0.3) S (0.18) C (3.4)	Fe (59) Cr (14.1)
	Bright	Ni (13.11) Mo (3.5) Mn (4) Si (1) O (0.77) P (0.8) S (0.08) C (3.6)	Fe (59) Cr (16)
$P16_1$		Cr (19.2) Mo (5.1) Mn (2.7) Si (1.7) O (1.5) P (0.85) S (0.87) C (3)	Fe (54) Ni (11.5)
$P1_2$	Central	Ni (14.5) Mo (3.5) Mn (8.7) Si (3.1) O (4.3) P (0.44) S (0.19) C (6.1)	Fe (46) Cr (14.3)
	Ring	Ni (14.6) Mo (3.2) Mn (2.7) Si (1.77) O (1.2) P (0.37) S (0.09) C (4.8)	Fe (56) Cr (14.6)
$P2_2$	Central	Mn (13.3) Si (4.3) O (6.7) P (0.44) S (0.1) C (5.9)	Fe (44) Cr (12.4) Ni (10.3) Mo (1.94)
	Ring	Ni (13.2) Mn (4.6) Si (2.2) O (2.1) P (0.35) S (0.07) C (4.7)	Fe (56) Cr (13.9) Mo (1.96)
$P3_2$		Ni (14) Mo (2.8) Mn (7) Si (2.6) O (2.6) P (0.43) S (0.14) C (5.5)	Fe (52) Cr (13.9)
$P4_2$	Central	Mn (9.3) Si (3.4) O (8) P (0.37) S (0.5) C (2)	Fe (51) Cr (13) Ni (10.3) Mo (0.9)
	Ring	Mn (3) Si (1.75) O (2.7) P (0.4) S (0.47) C (1.7)	Fe (62) Cr (14.9) Ni (12.5) Mo (0.94)
$P5_2$	Small	Mo (3.4) Mn (3.7) Si (1.9) O (2.2) P (0.4) S (0.18) C (3.6)	Fe (56) Cr (15) Ni (12.6)
	Large	Mo (5.6) Mn (7.5) Si (1.5) O (1) P (0.36) S (0.7) C (4)	Fe (53) Cr (14) Ni (11.9)
$P1_3$		Mn (18.6) Si (11.5) O (30.4) P (0.7) S (0.4) C (5.9)	Fe (16.8) Cr (11) Ni (3.6) Mo (2.27)
$P2_3$	Zone 1	Mo (5.7) Mn (20) Si (11.6) O (30) P (1.1) S (5.7) C (4.6)	Fe (11.75) Cr (9) Ni (2.2)
	Zone 2	Mo (4) Mn (15) Si (4.2) O (12.6) P (0.7) S (3.6) C (4)	Fe (31) Cr (15.5) Ni (7.3)
	Zone 3	Cr (18.5) Mo (3) Mn (18.5) Si (1.3) O (4.6) P (1.3) S (1.8) C (5.8)	Fe (49) Ni (11)
P1'	Zone 1	Mo (7.1) Mn (9.6) Si (6.6) O (11.7) P (1.25) S (5.4) C (3)	Fe (20.4) Cr (10.5) Ni (5.8)
	Zone 2	Cr (17.2) Mo (7.2) Mn (2.6) Si (1.8) O (3.7) P (1.1) S (1.8) C (3.45)	Fe (38) Ni (9.5)

	Zone 3	Mo (8.4) Mn (12.5) Si (8.1) O (15.8) P (2.3) S (5.5) C (3.9)	Fe (19) Cr (10.5) Ni (5.3)
P2'	Zone 1	Cr (18.9) Mo (7.2) Mn (4.2) Si (1.3) O (2.3) P (1.8) S (1.4) C (3.5)	Fe (47) Ni (12.4)
	Zone 2	Cr (19.6) Ni (13.8) Mo (7.3) Mn (2.9) Si (1.2) O (2.2) P (1.9) S (1.3) C (3.8)	Fe (48)
	Zone 3	Mo (4.7) Ni (14.1) Mn (2.7) Si (1.2) O (2.4) P (1) S (0.6) C (4)	Fe (54) Cr (16.5)
P3'	Zone 1	Cr (18.4) Mo (5.7) Mn (3.7) Si (0.9) O (2) P (1.8) S (1) C (3.7)	Fe (53) Ni (11.8)
	Zone 2	Cr (19.6) Mo (4.4) Mn (2.2) Si (0.9) O (2) P (1) S (0.6) C (3.2)	Fe (56) Ni (12.4)
	Zone 3	Cr (18) Mo (5.4) Mn (2.3) Si (0.9) O (2.6) P (1.7) S (0.8) C (5.5)	Fe (53) Ni (11.3)
P4'	Zone 1	Cr (17) Ni (16.5) Mo (4.9) Mn (3) Si (0.9) O (1.5) P (0.9) S (0.4) C (4.3)	Fe (52)
	Zone 2	Ni (14) Mo (7.6) Mn (7.1) Si (0.9) O (1.8) P (1) S (1.1) C (4.5)	Fe (46) Cr (16.2)
	Zone 3	Ni (14.2) Mo (6.9) Mn (5.2) Si (0.9) O (1.6) P (1) S (0.7) C (4)	Fe (48) Cr (16.8)
P5'	Dark	Mo (3.6) Mn (5.6) Si (2.1) O (5.7) P (0.44) S (0.4) C (7.1)	Fe (48) Cr (14.6) Ni (13.6)
	Bright	Mo (3.7) Mn (2.8) Si (0.9) O (2.1) P (0.36) S (0.15) C (6.2)	Fe (53) Cr (14.5) Ni (15.9)

S3. Finite element simulation geometry and mesh

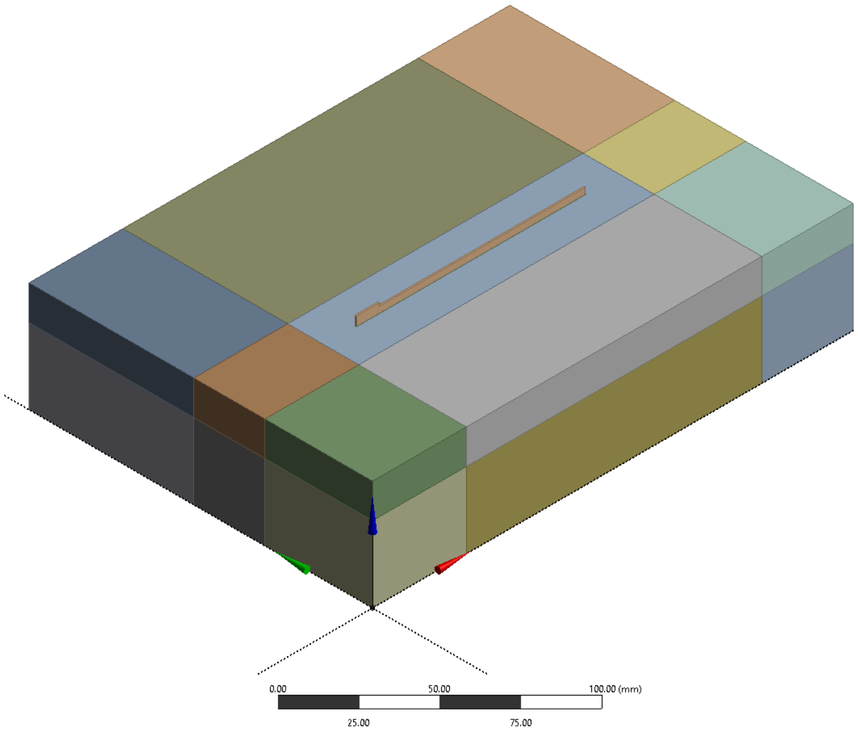

Figure S5: The simulated geometry for the LMD of the 3-layer 316LSS wall of Fig. 1. *This geometry includes the baseplate of 200 x 150 x 40 mm^3 that was not shown in Fig. 1. The substrate and the baseplate have been combined to form a thicker substrate of height 48 mm. The different colors show the domain partitioning that has been performed in order to generate a conformal mesh in Fig. S6; the domain partitioning is not related to the geometry of the baseplate and substrate. Gmsh v4.8.3 (https://gmsh.info/) and Microsoft Word have been used to prepare this image.*

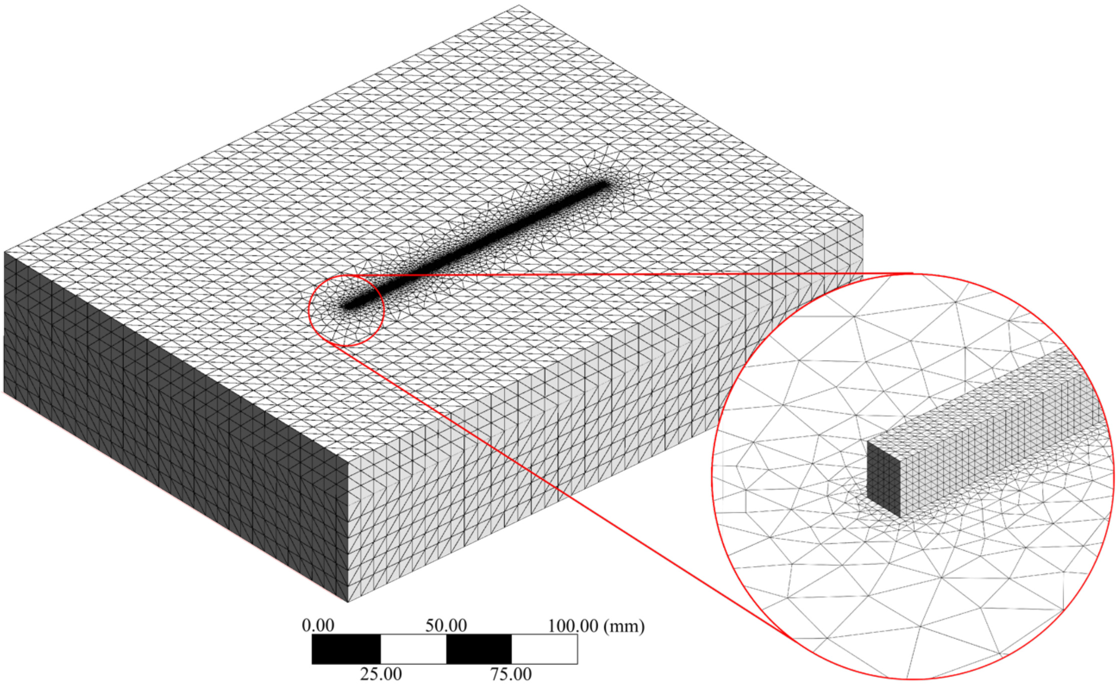

Figure S6: The meshed geometry for the LMD of the 3-layer 316LSS wall of Fig. S5. *This is a mesh at an instant where the third layer is being built. The substrate/baseplate is mainly made of a structured mesh with 6 mm seeds and the wall is made up of a structured mesh with 0.1 mm seeds. A portion of the substrate/baseplate has an unstructured mesh that allows transitioning from the 0.1 mm seeds of the wall to the 6 mm seeds of the remainder of the substrate/baseplate. All elements are tetrahedral with quadratic interpolation (P2). Gmsh v4.8.3 (https://gmsh.info/) and Microsoft Powerpoint have been used to prepare this image.*

S4. Phase stability analysis

This analysis is performed using the Thermo-Calc software version 2019b with the TCFE9 database to understand which phases are the most stable (i.e., lowest Gibbs free energy) equilibrium phases that could exist in the liquid and solid states in 316LSS. An elimination procedure (methods) is followed to gain insight on lesser stable phases that would not manifest in equilibrium but they could manifest during a non-equilibrium process such as LMD.

For these calculations, the 316LSS powder composition in atomic percent (at. %) is used (Table S2). For the 316LSS powder, the theoretical maximum oxygen composition that can be induced

during the inert gas atomization process is 0.05 wt.%, as reported by Höganäs[1]; this value has been used in the thermodynamic calculations.

Table S2: 316L powder composition in wt. % and at. %

Element	Cr	Ni	Mo	Mn	Si	C	P	S	O	Fe
wt.%	16.9	12.7	2.5	1.5	0.7	0.011	0.015	0.005	0.05	Bal.
at. %	18.063	12.025	1.448	1.517	1.385	0.051	0.027	0.009	0.174	Bal.

The "elimination" procedure for phase stability analysis is as follows:

1) Identify the most stable phases (lowest Gibbs free energy) that co-exist at a given temperature.
2) Eliminate a phase that is not γ-austenite or LA to reveal the next most stable phase.
3) Repeat 2 until either γ-austenite or LA remain.

The phase stability analysis has first been performed at (i) 1773 K and (ii) 873 K, which is below the solidus (< 1400 °C) but above the room temperature in order to limit exaggerated extrapolations of Gibbs free energy from high temperatures to room temperature by the Thermo-Calc software.

At 1773 K (1500 °C), two stable equilibrium phases are present in the liquid[2]: (i) Liquid A (LA) – molten 316LSS and (ii) Liquid B (LB) – Mn-Si-O rich with a composition close to $MnSiO_3$ having trace amounts of Fe and S. LA has a significantly higher volume fraction than LB. The process of elimination at 1773 K is shown as a sequence below:

$$\text{Sequence 1: LA + LB} \rightarrow \text{LA} + SiO_2 \rightarrow \text{LA} + MnSiO_3 \rightarrow \text{L1},$$

where the arrows indicate the elimination sequence and the eliminated phases are shown in blue. When the same elimination procedure is also performed at 1723 K (1450 °C) and 1873 K (1600 °C) as well as for different oxygen content (varying it from 0.05 wt. % to 0.1 wt. % to account for oxygen addition during LMD), it reveals the same sequence but with different phase fractions.

At 873 K (and lower temperatures), the most stable phases are γ-austenite, $\overline{M}_6C$ ($\overline{M}$ = Mo, Fe and Cr in descending wt. %), $MnSiO_3$ and M-S (M = mainly Mn, then Cr and Fe in descending wt. %). The elimination sequence is shown below:

$$\text{Sequence 2: } \gamma + \overline{M}_6C + \text{M-S} + MnSiO_3 \rightarrow \gamma + \overline{M}_6C + \text{M-S} + SiO_2$$
$$\rightarrow \gamma + \overline{M}_6C + \text{M-S} + MnO \rightarrow \gamma + \overline{M}_6C + \text{M-S} + Mn_2O_3 \rightarrow \gamma + \overline{M}_6C + \text{M-S} + MnO_2$$
$$\rightarrow \gamma + \overline{M}_6C + \text{M-S} \rightarrow \gamma + \overline{M}_6C + FeSO_4 \rightarrow \gamma + \overline{M}_6C$$

$\rightarrow$ the carbide is successively replaced by $\overline{M}_{23}C_6$ ($\overline{M}$ = Cr, Mo and Fe), $\overline{M}_7C_3$ ($\overline{M}$ = Cr, Mo and Fe), $\overline{M}_2C$ ($\overline{M}$ = Mo and Cr), $\overline{M}_3C$ ($\overline{M}$ = Cr and Fe) and $Cr_3C_2 \rightarrow \gamma$

Changing the oxygen content from 0.05 wt. % to 0.1 wt. % did not reveal any differences in sequence 2, although the phase fractions of the different phases were different. Completely removing oxygen results in the initial most stable composition to be $\gamma + \overline{M}_6C$ + M-S. Furthermore, successive elimination of the most stable non-austenite phases is the same as in sequence 2.

Successive elimination of M-S and $\overline{M}_6C$, reveal other carbides until only γ-austenite is left. Now, the TEM analysis has shown the presence of many non-oxide inclusions that are either intermetallics or richer in P along with S and/or C in comparison to the surrounding matrix, which implies that the elimination sequences 1 and 2 do not capture all the phases that could form during highly non-equilibrium processes such as LMD, SLM or inert gas atomization. Therefore, we only consider the phases occurring in the first step of sequence 1 and 2.

Finally, in the thermodynamic analysis performed in the main article, $\overline{M}_6C$ is not considered because of the following reasons: (i) $MnSiO_3$ and M-S are the most stable oxide and non-oxide phases that can exist with γ-austenite; this is supported by the Ellingham diagram[3] and the phase stability analysis shows that the Gibbs free energy of M-S (-150.8 kJ/mol at 873 K) is lower than that of $\overline{M}_6C$ (-44.9 kJ/mol at 873 K), (ii) M-S rich zones are present in many non-oxide inclusions in L1 – L5, and they have been reported to exist in different cooling studies for 316LSS[2,4–7], (iii) TEM observations are inconclusive on the presence/precise composition of carbides, and (iv) neglecting $\overline{M}_6C$ does not affect the composition and phase fraction of M-S and $MnSiO_3$.

References:

1. Höganäs portfolio for additive manufacturing (https://www.hoganas.com/globalassets/download-media/sharepoint/brochures-and-datasheets---all-documents/additive-manufactoring_hoganas-additive-standard-portfolio_2853hog.pdf).
2. Wakoh, M., Sawai, T. & Mizoguchi, S. Effect of S Content on the MnS Precipitation in Steel with Oxide Nuclei. *ISIJ International* **36**, 1014–1021 (1996).
3. Ellingham, H. J. T. Reducibility of oxides and sulphides in metallurgical processes. *Journal of the Society of Chemical Industry* **63**, 125–160 (1944).

4. Deng, P. *et al.* The origin and formation of oxygen inclusions in austenitic stainless steels manufactured by laser powder bed fusion. *Additive Manufacturing* 101334 (2020) doi:10.1016/j.addma.2020.101334.
5. Kim, H. S., Lee, H.-G. & OH, K.-S. Precipitation behavior of MnS on oxide inclusions in Si/Mn deoxidized steel. *Metals and Materials* **6**, 305–310 (2000).
6. Kim, H. S., Lee, H.-G. & Oh, K.-S. MnS precipitation in association with manganese silicate inclusions in Si/Mn deoxidized steel. *Metall Mater Trans A* **32**, 1519 (2001).
7. Tanaka, Y., Pahlevani, F., Moon, S.-C., Dippenaar, R. & Sahajwalla, V. In situ characterisation of MnS precipitation in high carbon steel. *Scientific Reports* **9**, 10096 (2019).
